# Personalized modeling of stress and blood pressure reactivity using mobile health data

**DOI:** 10.1038/s44184-026-00210-9

**Published:** 2026-05-08

**Authors:** Ali Kargarandehkordi, Aditi Jaiswal, Agnik Banerjee, Yang Qian, Christopher R. Slade, Yinan Sun, Tanvir Islam, Hiwot Belay Tadesse, Alexander Kostrinsky-Thomas, Chanhyun Park, Urmimala Sarkar, Elaine C. Khoong, Nhung Nguyen, Xuhai “Orson” Xu, Kristina T. Phillips, Roberto M. Benzo, Adrian Aguilera, Haopeng Zhang, Finale Doshi-Velez, Peter Washington

**Affiliations:** 1 Department of Information and Computer Sciences, University of Hawaiʻi at Mānoa, Honolulu, HI USA; 2 Division of Clinical Informatics and Digital Transformation (DoC-IT), Department of Medicine, University of California, San Francisco, CA USA; 3 Department of Computer Science, University of Maryland, College Park, MD USA; 4 Department of Computer Science, Brigham Young University–Hawaiʻi, Laie, HI USA; 5 Division of Cancer Prevention and Control, Department of Internal Medicine, College of Medicine, The Ohio State University Comprehensive Cancer Center, The Ohio State University Wexner Medical Center, Columbus, OH USA; 6 John A. Paulson School of Engineering and Applied Sciences, Harvard University, Cambridge, MA USA; 7 Department of Medicine, University of California, San Francisco, CA USA; 8 Health Outcomes Division, College of Pharmacy, The University of Texas at Austin, Austin, TX USA; 9 Division of General Internal Medicine, Zuckerberg San Francisco General Hospital, University of California, San Francisco, CA USA; 10 Division of General Internal Medicine, Department of Medicine, University of California, San Francisco, CA USA; 11 Department of Biomedical Informatics, Columbia University, New York, NY USA; 12 Center for Integrated Health Care Research, Kaiser Permanente Hawaiʻi, Honolulu, HI USA; 13 Department of Health Systems Science, Kaiser Permanente Bernard J. Tyson School of Medicine, Pasadena, CA USA; 14 School of Social Welfare, University of California, Berkeley, Berkeley, CA USA

**Keywords:** Biomarkers, Computational biology and bioinformatics, Physiology, Psychology, Psychology

## Abstract

Psychological stress is a key driver of short-term blood pressure (BP) elevations and cardiovascular risk, yet its moment-to-moment impact in daily life remains difficult to predict. In this longitudinal observational study, we collected multimodal data from 20 adults with self-reported hypertension, including continuous wearable-derived heart rate and activity, ecological momentary assessment (EMA) stress ratings, and ambulatory BP measurements in free-living conditions. The dataset comprised 3694 EMA responses and 3812 BP measurements collected over approximately four weeks per participant (mean 24.1 ± 8.5 days). We evaluated whether participant-specific (“personalized”) models outperform a single pooled population model. Two prediction tasks were examined: (i) prediction of near-term BP elevations from wearable signals and stress EMA responses and (ii) prediction of self-reported stress from wearable signals and BP. Across both tasks, personalized models consistently improved predictive performance. For BP prediction, personalized models achieved a mean validation AUROC of 0.803, exceeding the population model by 0.235, while for stress prediction they achieved a mean validation AUROC of 0.849, exceeding the population model by 0.208. These findings suggest that personalized wearable-based models can capture individual patterns of stress and BP dynamics, with direct implications for precision mental health assessment and just-in-time adaptive intervention design in future work.

## Introduction

Real-time artificial intelligence (AI) that forecasts short-horizon adverse events from passive data streams such as wearables can enable just-in-time adaptive interventions that are triggered through passive data streams reflecting complex mental states^1,2^. As a stride towards this eventual goal, we sought to build AI models to predict two intervenable health outcomes using patient-generated data: stress surges and BP elevations. We choose these two prediction targets because of their suitability for digital intervention: acute psychological stress can raise BP and elevate near-term cardiovascular risk, while stress-driven behaviors compound that risk, making timely detection actionable^3^. However, real-time prediction in the wild remains challenging compared to lab-collected data^4,5^.

A central obstacle when developing real-time detection models is inter-individual heterogeneity: population (pooled) models tend to average away person-specific baselines, diurnal rhythms, and stimulus–response patterns^6–8^. Personalized AI, or the training and evaluating of a separate model per participant, can potentially better capture these idiosyncrasies and align risk estimates to each person’s physiological range and behavior^8–10^. This possible increase in precision has the potential to reduce false alarms (mitigating alert fatigue) and support operating points matched to individual risk tolerance and goals^11–15^.

In order to study the paradigm of personalized AI for prediction of challenging-to-model high-frequency repeat adverse health events, we conducted a prospective 4-week study of 20 adults with self-reported hypertension, where we collected BP via the Omron HeartGuide, heart rate and steps via Fitbit, and app-based ecological momentary assessments (EMAs) of self-reported stress labels collected in daily life. We engineered a shared feature set (multi-scale window statistics, interactions, and time-of-day/week context), trained participant-specific ensembles combining gradient-boosted trees and bidirectional LSTM with attention, and compared them with a single global model trained on pooled data using each participant’s training split.

We evaluated two prediction tasks with distinct translational aims: (1) high-stress prediction from Fitbit and BP data and (2) prediction of elevated BP from Fitbit data and self-reported stress, with the knowledge that self-reported stress can potentially be replaced with non-invasive stress biomarkers in future work. These tasks are intended to evaluate predictive relationships among behavioral and physiological signals collected in daily life rather than to establish causal directionality between stress and BP dynamics. In this study, stress primarily refers to subjective psychological stress, as measured by participants’ EMA self-reports in daily life. This work is designed to provide feasibility data to support the future development of just-in-time adaptive interventions (JITAI) that are triggered by AI models operating on wearable and EMA data to predict the value of the “tailoring variable”.

## Results

### Participant characteristics and overall engagement

We analyzed data from 20 adults with hypertension followed for four weeks (mean 24.1 ± 8.5 days), yielding 3812 BP measurements (mean 201 ± 112 per participant) and 3694 EMA stress responses (mean 185 ± 123 per participant) across the cohort. The 20 participants are described in Table 1. Because the two prediction tasks required sufficient positive events at the participant level, the evaluable sample differed by analysis. For the elevated BP prediction task, 19 participants contributed participant-level model results; P17 was excluded from this task because of insufficient elevated BP labels. For the high-stress prediction task, 16 participants contributed participant-level model results; P17, P32, P33, and P40 were excluded because of insufficient high-stress labels. These task-specific exclusions also apply to the corresponding participant-level comparison plots and interpretability summaries.

**Table 1:** Participant demographics (ID, sex, age, and race/ethnicity)

IDs	Sex	Age	Race/Ethnicity
P10	Male	56	White
P15	Female	38	Native Hawaiian
P16	Male	22	Fijian
P17	Female	54	White
P18	Male	64	Native Hawaiian
P20	Male	34	Prefer not to state
P22	Male	33	Guamanian or Chamorro
P23	Female	42	Native Hawaiian
P24	Male	23	Filipino
P25	Female	49	Filipino
P26	Male	38	White
P30	Female	64	Chinese
P31	Male	33	White
P32	Male	46	Japanese
P33	Male	32	White
P34	Male	26	Filipino
P35	Female	35	Native Hawaiian
P36	Female	31	Chinese
P39	Female	45	Japanese
P40	Female	43	Native Hawaiian

“High stress” was defined a priori as EMA ratings greater than or equal to 6 on a 0-10 scale (12.7% of EMAs; mean 23.4 ± 39.5 per participant). For BPs, standard clinical thresholds (systolic BP > 130 mmHg or diastolic BP > 80 mmHg) were used for the majority of participants. For a subset of participants whose BP distributions produced severely imbalanced labels, participant-specific thresholds derived from each individual’s BP distribution were used to obtain a relatively balanced binary classification task for that individual. Depending on the participant’s observed BP distribution, these thresholds could be either higher or lower than the standard clinical thresholds. Accordingly, for these participants the elevated BP label represents a relative within-person BP elevation rather than a clinical hypertension threshold. The cohort included 11 men and 9 women, mean age 40.4 ± 12.3 years (range 22–64), with self-identified race/ethnicity distributions as follows: White (25%), Native Hawaiian (25%), Filipino (15%), Chinese (10%), Japanese (10%), Fijian (5%), and Guamanian/Chamorro (5%); one participant declined to disclose. Participant IDs and demographics appear in Table 1. Between-participant elevated BP prevalence varied (e.g., 5.1% for ID 17 to 22.1% for ID 30). Participants contributed, on average, 287 ± 89 labeled observations (time-aligned BP readings with derived features).

### Elevated BP prediction

We evaluated prediction of near-term BP elevation using wearable inputs (Fitbit heart rate and step counts) and stress EMAs to emulate passive, non-invasive monitoring. We sought to evaluate whether routine biosignals and perceived stress alone can forecast impending elevations without a contemporaneous cuff measurement. Personalized ensembles (XGBoost plus bidirectional LSTM with attention) were compared with a single pooled (global) model using the pre-specified per-participant split.

Table 2 reports per-participant performance and the gain from personalization. Personalized models consistently outperformed the pooled-global baseline (macro-average AUROC 0.803 vs 0.568; change in AUROC + 0.235), while participant-level sensitivity and specificity varied widely, consistent with heterogeneity in individual stress–BP coupling and daily activity context. For each participant, the final model configuration was selected based on AUROC computed on the validation partition, which also served as the evaluation partition because of the limited amount of participant-specific data available. The term *Best Pipeline* refers to the model (XGBoost-only, BiLSTM-only, or ensemble) that achieved the highest validation AUROC for that participant. The corresponding final hyperparameters, attention mechanisms, and ensemble weights are reported in Supplementary Tables 1-2. Model selection was performed across multiple architectures of varying complexity (XGBoost, BiLSTM, and ensemble), enabling comparison between simpler and more expressive models under identical temporal splits. Notably, substantial variability in selected architectures and ensemble weights was observed across participants, indicating that no single model configuration consistently outperformed others.

**Table 2:** Participant-level performance metrics for personalized and global elevated BP prediction models

ID	Elevation Proportion (train set)	Elevation Proportion (validation set)	Personalized AUROC	Global AUROC	Best Pipeline	Optimal Thr.	Sensitivity	Specificity
P10	87.10%	20.83%	0.814 ± 0.078	0.695 ± 0.093	P-LSTM	0.40	0.60 ± 0.16	0.90 ± 0.05
P15	87.81%	68.10%	0.761 ± 0.052	0.644 ± 0.052	P-LSTM	0.20	0.82 ± 0.04	0.67 ± 0.08
P16	50.91%	42.31%	0.736 ± 0.070	0.547 ± 0.088	P-Ensemble	0.52	0.63 ± 0.11	0.76 ± 0.08
P18	93.52%	88.46%	0.756 ± 0.119	0.591 ± 0.220	P-LSTM	0.53	0.27 ± 0.09	1.00 ± 0.00
P20	74.74%	62.50%	0.967 ± 0.044	0.772 ± 0.124	P-LSTM	0.36	1.00 ± 0.00	0.83 ± 0.16
P22	89.31%	79.17%	0.752 ± 0.098	0.502 ± 0.116	Pl-Ensemble	0.47	0.92 ± 0.04	0.50 ± 0.17
P23	8.38%	34.48%	0.919 ± 0.055	0.424 ± 0.112	P-Ensemble	0.46	0.90 ± 0.10	0.89 ± 0.07
P24	85.31%	73.17%	0.655 ± 0.090	0.491 ± 0.106	P-Ensemble	0.39	0.57 ± 0.09	0.82 ± 0.12
P25	58.08%	75.64%	0.795 ± 0.055	0.768 ± 0.054	P-Ensemble	0.50	0.64 ± 0.06	0.95 ± 0.05
P26	82.54%	25.95%	0.632 ± 0.060	0.600 ± 0.058	P-LSTM	0.48	0.47 ± 0.09	0.78 ± 0.04
P30	76.51%	78.48%	0.757 ± 0.040	0.697 ± 0.049	P-Ensemble	0.61	0.57 ± 0.04	0.89 ± 0.06
P31	26.20%	33.93%	0.749 ± 0.048	0.572 ± 0.059	P-LSTM	0.46	0.82 ± 0.06	0.62 ± 0.06
P32	61.76%	46.43%	0.695 ± 0.104	0.477 ± 0.120	P-Ensemble	0.75	0.92 ± 0.07	0.27 ± 0.12
P33	85.48%	92.11%	0.778 ± 0.102	0.690 ± 0.140	P-LSTM	0.39	0.60 ± 0.08	1.00 ± 0.00
P34	50.00%	66.67%	0.835 ± 0.159	0.335 ± 0.189	P-Ensemble	0.50	0.51 ± 0.22	1.00 ± 0.00
P35	96.97%	96.79%	0.716 ± 0.131	0.576 ± 0.152	P-Ensemble	0.43	0.84 ± 0.02	0.71 ± 0.19
P36	61.17%	71.43%	0.950 ± 0.069	0.720 ± 0.207	P-LSTM	0.54	0.80 ± 0.14	1.00 ± 0.00
P39	78.14%	71.43%	1.000 ± 0.000	0.400 ± 0.251	P-Ensemble	0.95	1.00 ± 0.00	1.00 ± 0.00
P40	11.43%	09.09%	0.978 ± 0.033	0.155 ± 0.133	P-LSTM	0.72	1.00 ± 0.00	0.95 ± 0.05

### High-stress prediction from wearables

We evaluated prediction of near-term high-stress episodes using Fitbit signals (heart rate, steps) and recent BP context derived around each EMA time stamp (±15 min systolic/diastolic statistics, a binary “any elevation” flag, and time since last BP reading/elevation). EMAs were binarized as high stress at greater than or equal to 6 on the 0–10 scale. Personalized models substantially outperformed the pooled global baseline (macro-average AUROC 0.849 vs 0.641; change in AUROC + 0.208). Consistent with heterogeneous stress reactivity and daily routines, sensitivity and specificity varied across individuals. Four participants (IDs 17, 32, 33, 40) had too few positive labels for stable stratified plots (e.g., SHAP panels) but were retained in aggregate metrics. Alongside AUROC/AUPRC, we report a high-recall threshold setting (Table 3).

**Table 3:** Per-participant comparison of personalized and global model performance for high-stress prediction

ID	High-stress Proportion (train set)	High-stress Proportion (validation set)	Personalized AUROC	Global AUROC	Best Pipeline	Optimal Thr.	Sensitivity	Specificity
P10	26.92%	23.15%	0.577 ± 0.059	0.692 ± 0.063	G-LSTM	0.49	1.00 ± 0.00	0.00 ± 0.00
P15	66.43%	25.47%	0.763 ± 0.054	0.742 ± 0.057	P-Ensemble	0.48	0.70 ± 0.09	0.75 ± 0.05
P16	20.45%	21.28%	0.778 ± 0.074	0.599 ± 0.098	P-Ensemble	0.41	0.79 ± 0.14	0.73 ± 0.07
P18	27.27%	12.00%	0.927 ± 0.080	0.685 ± 0.185	P-Ensemble	0.53	0.96 ± 0.18	0.77 ± 0.09
P20	44.44%	24.78%	0.699 ± 0.059	0.706 ± 0.060	G-LSTM	0.44	0.78 ± 0.08	0.60 ± 0.06
P22	2.78%	4.17%	0.880 ± 0.098	0.553 ± 0.229	P-Ensemble	0.47	0.88 ± 0.33	0.72 ± 0.07
P23	17.22%	6.52%	0.872 ± 0.079	0.540 ± 0.128	P-LSTM	0.67	0.94 ± 0.23	0.72 ± 0.07
P24	11.63%	4.49%	0.928 ± 0.028	0.429 ± 0.150	P-Ensemble	0.50	0.98 ± 0.12	0.88 ± 0.03
P25	73.63%	80.56%	0.835 ± 0.058	0.725 ± 0.077	P-Ensemble	0.57	0.82 ± 0.05	0.86 ± 0.10
P26	19.81%	9.62%	0.866 ± 0.074	0.753 ± 0.090	P-LSTM	0.50	0.90 ± 0.10	0.74 ± 0.05
P30	32.66%	20.63%	0.750 ± 0.055	0.564 ± 0.064	P-LSTM	0.60	0.61 ± 0.10	0.81 ± 0.04
P31	12.45%	10.00%	0.985 ± 0.012	0.880 ± 0.039	P-Ensemble	0.58	1.00 ± 0.00	0.91 ± 0.03
P34	15.38%	42.86%	1.000 ± 0.000	0.578 ± 0.262	P-Ensemble	0.49	0.98 ± 0.15	0.75 ± 0.24
P35	9.20%	00.52%	1.000 ± 0.000	0.448 ± 0.037	P-Ensemble	0.50	0.59 ± 0.49	0.99 ± 0.01
P36	25.53%	66.67%	0.819 ± 0.067	0.401 ± 0.106	P-Ensemble	0.33	0.70 ± 0.09	0.93 ± 0.06
P39	2.11%	00.66%	0.908 ± 0.024	0.967 ± 0.015	G-LSTM	0.76	0.61 ± 0.49	0.76 ± 0.03

In the stress prediction task, BP measurements were included as contextual physiological features rather than primary stress biomarkers. While BP is not typically used as a direct indicator of acute stress in psychophysiological literature, it reflects cardiovascular state and may capture downstream or concurrent physiological responses associated with stress. Importantly, our objective was not to establish causal relationships between stress and BP, nor to determine whether BP elevations are a cause or consequence of stress. Rather, we focus on engineering predictive models that leverage available multimodal signals to passively infer stress states in real-world settings. Accordingly, BP is treated as an informative contextual signal within a predictive framework rather than as a mechanistic marker of stress physiology. This design reflects a pragmatic, multimodal modeling approach rather than a causal or mechanistic model of stress physiology.

**Fig. 1:**
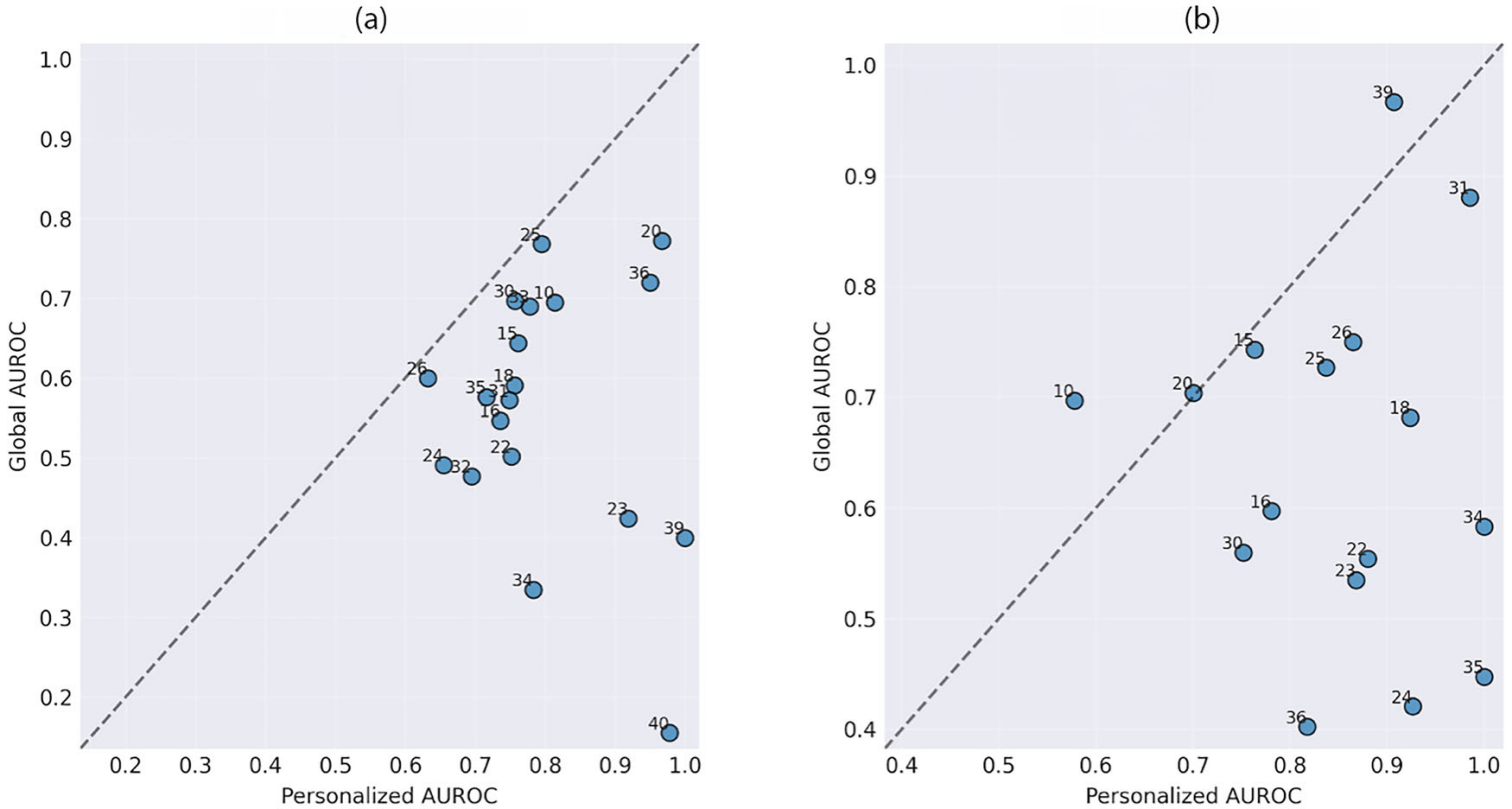
Participant-level AUROC for personalized vs pooled-global models across the three tasks. Most points lie below the parity line (y = x), indicating consistent benefit of personalization: for elevated BP prediction, 19/19 improved (panel **a**), and 13/16 improved for high-stress prediction (panel **b**; four participants lacked sufficient high-stress positives and were omitted).

Fig. 1a summarizes elevated BP prediction: all 19 participants benefited from personalization (19/19 points fall below the y = x line), and none favored the global model. Participant 17 was excluded due to insufficient elevated BP labels.

Figure 1b summarizes high-stress prediction: 13/16 participants benefited from personalization (points below y = x; 3/16 favored the global model). Participants 17, 32, 33, and 40 were excluded due to insufficient high-stress labels.

For a small subset of participants (e.g., P39 in BP prediction and P34–P35 in stress prediction), AUROC values of 1.000 with zero bootstrap variance were observed. Upon further inspection, these cases corresponded to participants with relatively small validation sets and highly separable class distributions.

### Feature importance and temporal dynamics

SHAP analyses of the XGBoost models (Supplementary Fig. 1 for elevated BP; Supplementary Fig. 2 for high-stress) show that short-horizon heart rate statistics, recent step dynamics, and stress-related features are consistently influential, with marked between-participant variability in both ranking and direction of effects. This heterogeneity aligns with, and helps explain, the gains from personalized modeling.

### Ablation analysis

To assess the relative contribution of different feature families, we performed per-participant grouped ablation experiments in which feature families were removed prior to retraining personalized models, revealing that the most-critical predictors varied across participants (Table 4). Step and stress features were most frequently identified as most critical overall. Other feature categories – cumulative features, heart rate features, ratios, and time features – were each most-critical for a single participant. In every case, removal of the most-critical family reduced discrimination relative to the full-feature baseline. Notably, least-impact does not necessarily imply worse performance than the baseline. In many participants, removal of the least-impact feature family increased AUROC, suggesting that some feature types may introduce noise for certain individuals. For instance, after removing cumulative features, AUROC improved from 0.752 to 0.869 for PID 22; from 0.835 to 0.945 for PID 34 after removing stress features; and from 0.716 to 0.811 for PID 35 after removing time features. Conversely, some participants experienced substantial declines, such as PID 15, whose AUROC decreased from 0.761 to 0.613 when stress features were removed (comprehensive results are available in Supplementary Table 3).

**Table 4:** Per-participant grouped ablation (most-critical)

ID	Group	AUROC Before Ablation	AUROC After Ablation	Optimal Threshold
P15	Stress features	0.761 ± 0.052	0.613 ± 0.059	0.49
P16	Stress features	0.736 ± 0.070	0.629 ± 0.085	0.46
P18	Time features	0.756 ± 0.119	0.813 ± 0.091	0.48
P20	Heart rate features	0.967 ± 0.044	0.917 ± 0.092	0.16
P22	Cumulative	0.752 ± 0.098	0.869 ± 0.061	0.50
P22	Step features	0.752 ± 0.098	0.884 ± 0.056	0.47
P23	Step features	0.919 ± 0.055	0.809 ± 0.098	0.36
P24	Step features	0.655 ± 0.090	0.539 ± 0.105	0.00
P25	Stress features	0.795 ± 0.055	0.675 ± 0.071	0.49
P31	Step features	0.749 ± 0.048	0.674 ± 0.058	0.53
P33	Step features	0.778 ± 0.102	0.510 ± 0.111	0.99
P34	Stress features	0.835 ± 0.159	0.945 ± 0.089	0.47
P35	Time features	0.716 ± 0.131	0.811 ± 0.055	0.30
P35	Ratios	0.716 ± 0.131	0.807 ± 0.063	0.49
P40	Ratios	0.978 ± 0.033	0.875 ± 0.112	0.42

For each participant, the feature family whose removal produced the highest difference in AUROC (compared to before ablation) among ablation runs is reported, together with the Youden-selected threshold. Feature families are defined as follows: *Heart rate features* (short-horizon heart rate statistics), *step features* (multi-scale step dynamics), *stress features* (EMA-derived stress summaries), *ratios* (e.g., HR/steps, stress-weighted HR), *cumulative* (short rolling sums/means), and *time features* (calendar/context indicators).

The least-impact feature family also varied considerably (Table 5). Time features were most often least-impact, followed by cumulative features, and heart rate for a few participants (comprehensive results are available in Supplementary Table 3). These findings demonstrate that feature importance is highly individualized, highlighting the utility of participant-level feature selection in personalized models.

**Table 5:** Per-participant grouped ablation (least-impact)

ID	Group	AUROC Before Ablation	AUROC After Ablation	Optimal Threshold
P10	Cumulative	0.814 ± 0.078	0.818 ± 0.091	0.38
P15	Step features	0.761 ± 0.052	0.717 ± 0.046	0.58
P18	Stress features	0.756 ± 0.119	0.759 ± 0.181	0.56
P20	Cumulative	0.967 ± 0.044	0.951 ± 0.059	0.36
P22	Stress features	0.752 ± 0.098	0.757 ± 0.085	0.85
P22	Ratios	0.752 ± 0.098	0.733 ± 0.105	0.50
P23	Cumulative	0.919 ± 0.055	0.909 ± 0.055	0.22
P24	Ratios	0.655 ± 0.090	0.692 ± 0.091	0.48
P26	Step features	0.632 ± 0.060	0.650 ± 0.057	0.33
P30	Step features	0.757 ± 0.040	0.718 ± 0.043	0.58
P31	Time features	0.749 ± 0.048	0.721 ± 0.051	0.31
P33	Ratios	0.778 ± 0.102	0.760 ± 0.105	0.34
P36	Cumulative	0.950 ± 0.069	0.950 ± 0.069	0.51
P36	Time features	0.950 ± 0.069	0.950 ± 0.069	0.55
P40	Time features	0.978 ± 0.033	0.978 ± 0.033	0.37

For each participant, the feature family whose removal produced the lowest difference in AUROC (compared to before ablation) among ablation runs is reported, together with the Youden-selected threshold.

### Latent space visualization and model interpretability

**Fig. 2:**
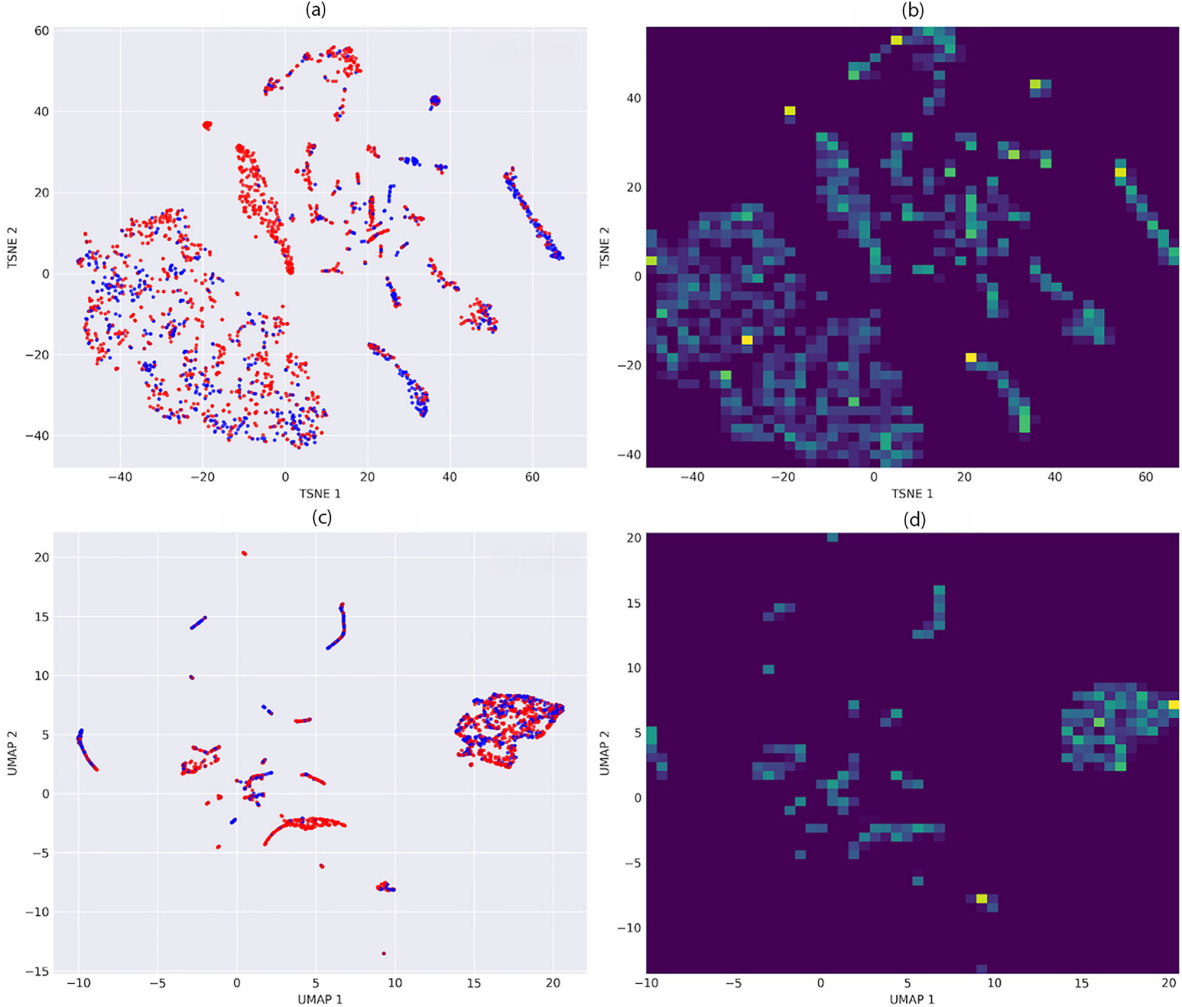
Class Separability Between Elevated and Non-Elevated BP Observations. t-SNE (**a**,**b**) and UMAP (**c**,**d**) embeddings of model-derived feature representations, colored by elevated BP status (red = elevation, blue = non-elevation; a,c) and shown as density maps (**b**,**d**).

Latent space projections using t-SNE and UMAP (Fig. 2) revealed clear separation between elevation and non-elevation events, with distinct participant-specific neighborhoods reflecting individual physiological signatures. Regions of overlap corresponded to ambiguous physiological states where classification was more difficult, while variations in point density reflected the class imbalance, with denser clusters for non-elevation events.

To assess the robustness of model performance over varying monitoring durations, we conducted additional temporal stability analyses (Supplementary Note 1; Supplementary Figs. 3–6).

## Discussion

We find that personalized models tend to outperform generalized models on two challenging prediction tasks: (1) high-stress prediction from Fitbit and BP data and (2) prediction of elevated BP from Fitbit data and self-reported stress, with the knowledge that self-reported stress can be replaced with non-invasive stress biomarkers in future work. We note that the two prediction tasks should therefore be interpreted as complementary predictive analyses of multimodal physiological and behavioral signals rather than causal tests of the relationship between psychological stress and BP elevation.

In the elevated BP prediction task using only wearables and stress (no prior BP inputs), personalized models achieved a mean AUROC of 0.803, outperforming the global model by +0.235 AUROC on average. For the high-stress prediction task, personalized models achieved a mean AUROC of 0.849, outperforming the pooled/global model (mean AUROC = 0.641) by +0.208 AUROC on average. Model explanations from both methods told a consistent story: SHAP (XGBoost) and attention weights (BiLSTM) highlighted the same signals (i.e., short-horizon heart rate patterns, recent step dynamics, and contemporaneous self-reported stress) as the most informative. Rather than capturing exercise per se, these features may reflect short-term physiological and behavioral changes, including transient increases in cardiovascular activity and contextual shifts in movement patterns that precede BP elevations. While such patterns are consistent with prior observations linking stress and cardiovascular activation, it is important to note that heart rate alone, without complementary measures such as heart rate variability, provides limited insight into underlying autonomic mechanisms, particularly in free-living settings where movement and environmental factors may confound physiological signals. In future work, the self-reported stress EMA can be replaced by an objective stress biomarker (for example, wearable skin conductance) integrated with the wrist-based physiology from devices such as the Fitbit. EDA has been shown in field and lab studies to correlate with sympathetic arousal and perceived stress (e.g., mean accuracy approximately 82.6% for stress detection in EDA wearable studies)^16^. An AI model that takes EDA, heart rate, and step data as inputs, calibrated per-participant over a few days, could potentially be used to passively anticipate elevated BP events and, in future work, inform just-in-time adaptive interventions (e.g., coping prompts, brief breathing exercises, or re-measurement of BP). However, it is important to note that no intervention was evaluated in this study, and the effectiveness, safety, and user acceptability of such intervention strategies remain to be established in future prospective trials. Previous digital interventions for BP control and stress/mental-health support (such as smartphone apps and remote monitoring for hypertension) have demonstrated the feasibility of app-supported behavioural change for hypertension management (see e.g., digital therapeutics in hypertension)^17^. Our proposed model extends this prior work by bringing in real-time sensing, personalization, and predictive modelling of both stress and BP, thereby enabling adaptive triggering of interventions rather than only retrospective monitoring or generic prompts.

In a small number of participants, the model achieved AUROC values of 1.000 with zero bootstrap variance. While this may appear to indicate perfect predictive performance, these cases were associated with relatively small validation sets and highly separable class distributions, where predicted probabilities for positive and negative instances did not overlap. As a result, bootstrap resampling yielded identical performance estimates across iterations. The findings for this small subset of participants should be interpreted with caution, as they likely reflect limited sample sizes and strong within-participant separability rather than true perfect generalization. Future work with longer monitoring periods and larger, more diverse cohorts is needed to assess the stability and generalizability of these personalized models.

In addition, an AI model that takes in ambulatory BP readings combined with wrist-based wearable data (such as heart rate and step count) could similarly enable passive prediction of elevated stress states without requiring real-time self-reports. In future implementations, such predictions could be used to inform just-in-time digital interventions (e.g., mindfulness prompts or coping strategy notifications). However, these applications remain speculative, as intervention strategies were not tested in this study. Prior digital interventions targeting stress control (for instance, smartphone-based stress-management apps and biofeedback interventions) have demonstrated feasibility and efficacy in reducing perceived stress^18,19^. By embedding the predictive model upstream of the intervention and tailoring thresholds to the user’s baseline, this approach extends prior work by shifting from reactive to proactive, individualized intervention delivery, potentially improving timing, adherence, and outcomes.

From a clinical and intervention design standpoint, the trade-off between sensitivity and specificity determines the type of digital intervention that an AI model can effectively support. A model tuned for high sensitivity but lower specificity, prioritizing the detection of nearly all potential BP elevations, would be suitable for preventive or low-burden interventions, such as mindfulness prompts, paced breathing, or short activity reminders, where the cost of a false alarm is minimal. Conversely, a model optimized for high specificity but lower sensitivity, triggering alerts only when confidence is high, would align with interventions carrying higher effort or clinical consequence, such as medication reminders, clinician notifications, or behavioral coaching sessions. This calibration flexibility allows systems to tailor intervention thresholds to the user’s context and tolerance for false positives. In our results (AUROC of approximately 0.80; specificity of approximately 0.80), the operating point that we use (decision threshold of 0.5) represents a practical middle ground, minimizing unnecessary alerts while maintaining reasonable sensitivity for timely detection. However, we could in theory tune the threshold of these personalized models to optimize sensitivity or specificity, depending on the type of digital intervention being created. Similar sensitivity–specificity trade-offs have been discussed in Li et al.^20^, where individualized thresholds were proposed to adapt AI-driven interventions for substance-use prediction based on each participant’s preferred balance between missed detections and alert burden.

To further contextualize these findings, we conducted grouped feature ablation analyses to quantify the impact of removing entire feature families on predictive performance. The results reinforce the interpretation that predictive signal is highly individualized: no single feature family consistently dominated across participants. Step dynamics and EMA-derived stress features were most frequently critical, suggesting that short-term behavioral context and subjective stress reports provide complementary predictive value in many individuals. However, in several participants, removing certain feature families (e.g., cumulative or ratio-based features) improved performance, indicating that some engineered features may introduce noise or redundancy depending on the individual. This heterogeneity highlights that model performance gains from personalization arise not only from model parameters but also from participant-specific relevance of feature groups. Importantly, these findings should be interpreted as evidence of predictive utility rather than mechanistic importance, as the feature set and observational design do not allow causal attribution of physiological drivers.

Despite promising predictive performance, a substantial gap remains between model performance and safe, effective clinical deployment. Translating these models into real-world systems requires careful evaluation of intervention timing, user burden, and potential unintended consequences. For example, false positive alerts may contribute to alert fatigue or increased anxiety, while false negatives may delay necessary interventions. These trade-offs were not quantified in the current study and warrant dedicated investigation in future trials.

Our study has several limitations. First, the cohort (*n* = 20; mean age approximately 40 years, maximum 64) has limited generalizability to older adults, particularly to those over 65 years of age in whom hypertension is most prevalent, and to subgroups with multimorbidity or pregnancy-related hypertension. Because the study involved intensive longitudinal monitoring of a relatively small cohort, the resulting personalized models may partially capture stable behavioral routines or measurement patterns rather than purely physiological mechanisms linking stress and BP changes. Accordingly, predictive performance estimates should be interpreted cautiously and require validation in larger and more diverse cohorts to evaluate the generalizability of these findings. Second, BP labels depended on adherence to a wrist-worn cuff, creating potential gaps between readings and introducing technique-sensitive measurement error associated with wrist positioning and user-initiated measurements in free-living conditions. Some elevations may therefore have been missed or misclassified. Third, although we included hour-of-day and day-of-week covariates, diurnal phenomena (e.g., morning surge, nocturnal dipping) were not systematically modeled. Because BP measurements were participant-initiated and therefore irregularly spaced, circadian BP variation could only be modeled indirectly through calendar features rather than through structured ambulatory BP monitoring schedules. Fourth, EMA completion varied across participants, and we did not incorporate objective stress proxies (e.g., electrodermal activity), which may have reduced label quality and physiologic coverage. Fifth, wearable-derived physiological signals were obtained from consumer-grade wearable sensors. Although such devices enable continuous monitoring in free-living environments, comparative validation studies have shown that wearable devices can differ substantially in their estimation of cardiovascular and autonomic signals in real-world settings^21,22^. Wearable-derived signals may also exhibit variability depending on motion, activity level, device placement, skin characteristics, and device-specific algorithms, introducing measurement uncertainty that may propagate into downstream predictive models and should therefore be considered when interpreting personalized physiological signals derived from consumer wearables. Additionally, heart rate changes measured by wearables may reflect both psychological stress responses and physiological arousal related to movement or exercise, limiting interpretation of wearable-derived physiological signals. Finally, the observational design precludes causal inference (unmeasured factors such as sodium intake, posture, or stimulants may confound associations), and we did not quantify the energy footprint of continuous on-device inference, which may constrain deployment of the LSTM component. An additional limitation is that, because of the relatively small amount of longitudinal data available for each participant, the temporal partition was used both for model selection and final performance estimation. This design reflects the exploratory nature of the present study and may produce optimistic performance estimates compared with evaluation on a completely independent test set. Future studies with larger longitudinal datasets should include separate training, validation, and external test partitions to provide more rigorous estimates of model generalizability. Moreover, per-participant selection of the LSTM architecture, ensemble weighting, and decision threshold was performed on the same validation data used for reporting, which could have inflated the benefit of the personalization over the generalized model.

Although heart rate variability (HRV) metrics such as RMSSD, SDNN, and LF/HF ratio are widely used in stress detection, they could not be reliably derived from the available wearable data. The Fitbit device provides averaged heart rate measurements rather than beat-to-beat interbeat intervals (IBI), which are required for accurate HRV computation. While Fitbit reports proprietary HRV estimates during resting periods, these are not available continuously and therefore are not suitable for real-time prediction tasks aligned with EMA and BP measurements. As a result, HRV-based features were not included in this study.

Future work should (i) broaden to demographically and clinically diverse cohorts and conduct prospective external validation; (ii) obtain denser BP labels via optimized prompting or cuffless sensors; (iii) enrich context with medication timing/adherence and common vasoactive exposures (e.g., decongestants, NSAIDs, caffeine/energy drinks), alongside sleep, posture, and dietary sodium; (iv) apply causal tools (e.g., Granger tests, counterfactual and mediation analyses) to separate stress- from activity-driven BP elevations; (v) optimize on-device inference (quantization/pruning/distillation) for sustained smartwatch deployment; (vi) embed predictions in just-in-time adaptive interventions, evaluated in micro randomized or randomized trials for effects on BP control, burden, and patient-reported outcomes; and (vii) evaluate these modeling approaches in larger and more diverse cohorts and include prospective validation.

In conclusion, this work demonstrates that wearable-derived biosignals, combined with minimal EMA input, can capture individualized patterns associated with BP fluctuations and perceived stress in free-living conditions. Personalization substantially enhances predictive performance. While these findings highlight the potential of wearable-based AI for personalized monitoring, further validation in larger and more diverse cohorts, as well as prospective interventional studies, is required to assess clinical utility and real-world impact. Additionally, the real-world implications of different operating points, including their impact on user burden, adherence, and clinical outcomes, were not evaluated in this study and remain an important direction for future work.

## Methods

### Study design and data collection

This study was conducted in accordance with the Declaration of Helsinki and all relevant institutional guidelines and regulations. The research protocol was reviewed and approved by the University of Hawaiʻi Institutional Review Board (protocol #2023-00130) and received additional approval through the University of Hawaiʻi Data Governance Process (request #DGP 231116-4). All participants provided written informed consent on paper during the intake session before any study activities began. The consent process informed participants about data collection procedures, confidentiality protections, potential risks, and their right to withdraw at any time without penalty.

We conducted a 4-week longitudinal study of 20 adults with a history of hypertension and elevated perceived stress. Participants wore a Fitbit device for continuous physiology (heart rate, step counts) and an Omron HeartGuide for intermittent, user-initiated BP measurements (approximately hourly when feasible). Participants were instructed to position the Omron device at heart level during BP measurements in accordance with manufacturer guidance to reduce posture- or motion-related measurement error. Perceived stress was captured via our custom-developed CardioMate app using EMA prompts delivered multiple times per day (median 6 entries/day per participant), yielding continuous biosignals aligned with time-stamped stress labels. EMA responses were treated as measures of subjective psychological stress. In contrast, wearable-derived signals such as heart rate and step counts were treated as physiological/contextual inputs that may reflect both psychological stress responses and activity-related signals. The current study did not explicitly distinguish exercise-related physiological activation from psychological stress, although activity features were included to provide contextual information during modeling. Heart rate variability (HRV) features were not included because the available wearable data did not provide continuous interbeat interval (IBI) signals required for reliable HRV estimation.

Hypertension status and current antihypertensive use were self-reported at intake; medication regimens were not standardized and are treated as potential confounders in our interpretation due to the personalized nature of our modeling. Participants reported several commonly prescribed antihypertensive and metabolic medications, including angiotensin receptor blockers (e.g., losartan, olmesartan), ACE inhibitors (lisinopril), calcium channel blockers (amlodipine, diltiazem), statins (atorvastatin), and antidiabetic medication (glipizide). Some participants also reported comorbid conditions including high cholesterol, diabetes or borderline diabetes, sleep apnea, asthma, anxiety disorders, and other chronic conditions. These variables were self-reported and were not incorporated as predictors in the modeling pipeline. The study protocol did not standardize which wrist was used for the BP device, and this information was not systematically recorded.

**Fig. 3:**
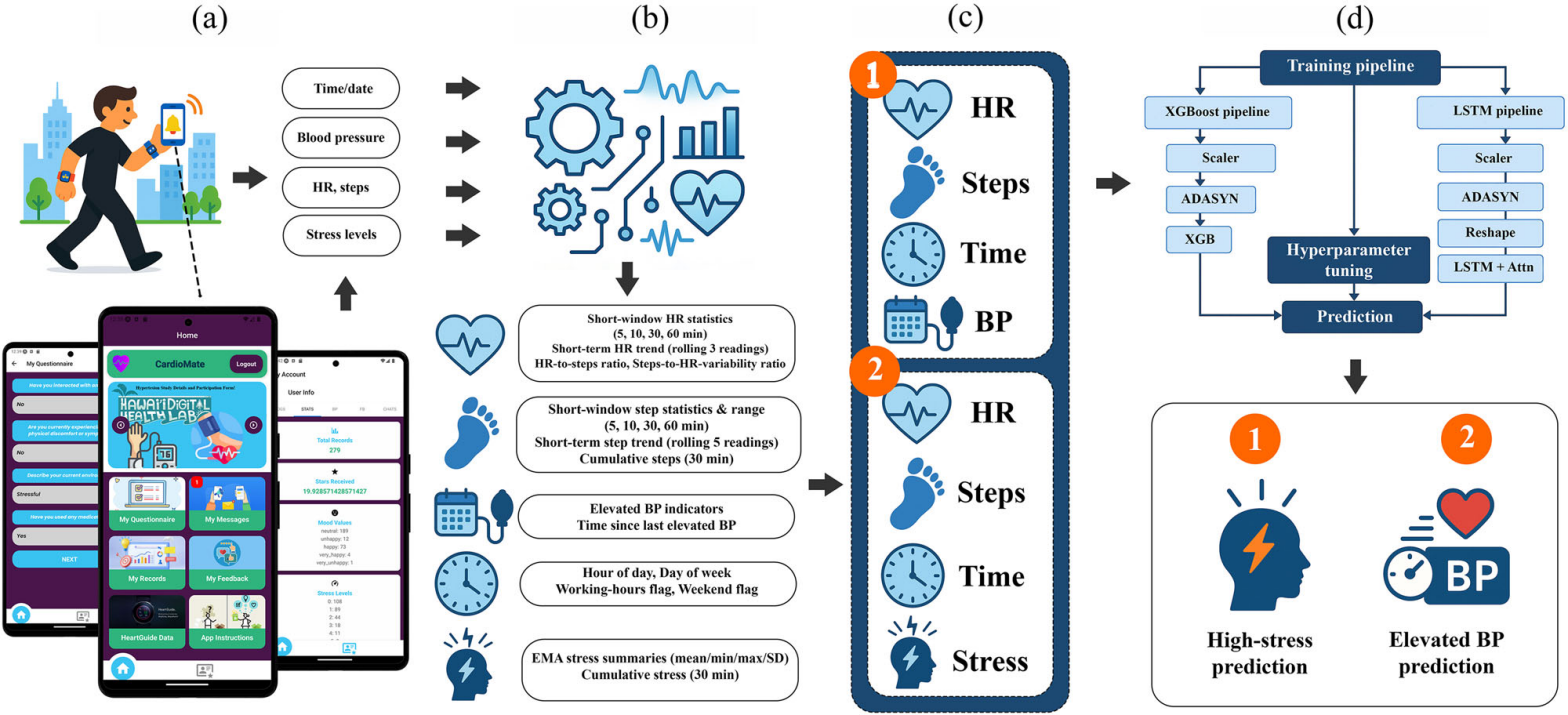
Data and modeling workflow. Inputs (**a**): Omron HeartGuide BP readings, Fitbit heart rate and step signals, and in-app EMA of stress (0–10 scale) collected from our custom CardioMate app are used as model inputs. Data streams are time-aligned and transformed into engineered features (**b**). Features are selected for each task (**c**). We evaluate two model families (**d**): XGBoost (with StandardScaler and ADASYN for class-imbalance) and a bidirectional long short-term memory (BiLSTM) with attention (scaling → optional ADASYN → sequence reshape → LSTM+attention). Hyperparameters are tuned with Keras Tuner and models are evaluated on validation splits (per-participant and global/pooled). Outputs include elevated BP prediction, stress prediction, and correlation analyses supporting interpretability.

The study protocol ensured participants maintained their usual routines, to capture naturalistic data such as stress responses and physiological changes, while minimizing participant burden. The combination of continuous objective biosensor data with subjective stress reports provided a comprehensive dataset for understanding the temporal dynamics between stress exposure and blood pressure changes. The overall data processing and model pipeline, from multi-source sensing to evaluation, is illustrated in Fig. 3.

### Data preprocessing

We defined an elevated BP label at reading t as

1 $${y}_{t}=1({{SBP}}_{t} > {\tau }_{{SBP}}\vee {{DBP}}_{t} > {\tau }_{{DBP}})$$

where the systolic and diastolic thresholds are denoted by ${\tau }_{{\rm{SBP}}}=130\,{\rm{mmHg}}$ and ${\tau }_{{\rm{DBP}}}=80\,{\rm{mmHg}}$. These cutoffs were applied for the majority of participants, reflecting standard clinical hypertension thresholds. However, for a small subset of participants whose BP distributions resulted in severely imbalanced labels (e.g., extremely few or extremely many elevated BP events), we instead applied a participant-specific cutoff, selected from each individual’s own BP distribution to obtain a minimally balanced classification problem. Depending on the participant’s distribution, this cutoff could be higher or lower than the standard 130/80 mmHg thresholds; for these participants the elevated-BP label therefore denotes a relative within-person elevation rather than a clinical hypertension threshold. Heart rate and step data were time‑sorted and aligned to each BP timestamp using an as‑of merge (nearest prior sample), and rolling statistics (mean, min, max, standard deviation) were computed for 5-, 10-, 30-, and 60-minute windows. Self‑reported stress entries from EMA responses were aggregated within a ± 15‑minute window around each BP reading to compute stress mean, min, max, and standard deviation. We then introduced simple interaction terms to capture relationships between physiological and behavioral signals: (2) represents the ratio between short-term heart rate and physical activity (steps), providing a proxy for relative cardiovascular load independent of movement. (3) reflects stress-weighted heart rate, capturing the interaction between perceived stress and physiological arousal. (4) represents the ratio of stress to activity, providing a contextual measure of perceived stress relative to concurrent physical activity.

2 $${HR}/{{Steps}}_{t}=\frac{{{hr}\_{mean}\_5\min }_{t}}{{{steps}\_{total}\_10\min }_{t}}+1$$

3 $${{stress}\_{weighted}\_{HR}}_{t}={{hr}\_{mean}\_5\min }_{t}\times {{stress}\_{mean}}_{t}$$

4 $${stress}/{{steps}}_{t}=\frac{{{stress}\_{mean}}_{t}}{s{{teps}\_{total}\_10\min }_{t}+1}$$

In addition, we computed a variability ratio ${\rm{steps\_std\_}}10\min /({\rm{hr\_std\_}}10\min +{10}^{-5})$ to characterize relative fluctuations in movement and cardiovascular signals. We also computed short rolling sums and means over temporal context (hour-of-day, day-of-week, working-hours flag, weekend), and time since the previous BP reading to reflect sampling gaps. These temporal context variables were included to partially capture diurnal variation in ambulatory BP. To reduce potential measurement artifacts and incomplete physiological context, we excluded terminal elevated BP rows that lacked subsequent measurements or surrounding wearable signals (e.g., the last elevated BP in a series). Finally, we removed each participant’s first day to minimize acclimation effects, early atypical wear and measurement anxiety that can transiently elevate readings and distort baseline physiology.

### Data splitting

We employed a temporal splitting strategy that preserved the sequential nature of the data. Data were grouped by calendar day. To preserve temporal ordering and avoid information leakage, participant-specific temporal splits were defined using a predefined number of training days determined before model fitting. The remaining observations formed a temporally separated held-out evaluation partition. Because the amount of longitudinal data varied substantially across participants, the duration of the training period was selected individually rather than using a fixed proportion of each participant’s data. Given the limited amount of participant-specific data available for personalized modeling, the temporally-later partition served both as the validation set for model selection and hyperparameter tuning and as the final evaluation set. In other words, we deliberately used only a train/validation split with no separate held-out test set due to the limited per-participant dataset size. Accordingly, the reported results should be interpreted as exploratory estimates of predictive performance rather than fully independent external test performance. In addition, all engineered features were computed using only historical information available prior to each prediction time point. No future observations were used in feature construction, ensuring strict temporal causality throughout the pipeline.

### Model architecture and training pipelines

We developed and evaluated two complementary modeling approaches for predicting stress-induced elevated BP: a personalized ensemble model and a pooled-global ensemble model. Both approaches combine a gradient boosting pipeline leveraging engineered features and a deep learning pipeline capturing temporal dependencies using attention mechanisms.

The first model utilized XGBoost, integrated within a comprehensive pipeline to address class imbalance through Adaptive Synthetic Sampling (ADASYN). The pipeline included three stages: (1) Standardization using the StandardScaler to normalize feature distributions, (2) ADASYN to generate synthetic minority class samples, and (3) XGBoost classification with dynamically computed class weights based on participant-specific elevated BP prevalence. Hyperparameter optimization was performed through a 3-fold cross-validated grid search, exploring multiple hyperparameter dimensions. For ADASYN, sampling strategies ranging from 0.6 to 0.75 were tested, with the number of neighbors fixed at 5. For XGBoost, we evaluated tree depths of 3, 5, and 7, learning rates of 0.01, 0.05, and 0.1, and ensemble sizes of 100, 150, and 200 estimators. The scale_pos_weight parameter was dynamically computed as the ratio of negative to positive samples, ensuring effective handling of class imbalance.

The second approach employed a sophisticated deep learning architecture to capture complex temporal dependencies in physiological time series. We used bidirectional LSTM (BiLSTM) layers to process sequential data in both forward and backward directions, allowing the model to capture both past and future context. The first BiLSTM layer consisted of 64–256 units (optimized through hyperparameter search) and was followed by batch normalization and dropout (0.2–0.5) for regularization. A second BiLSTM layer (32–128 units) further refined the temporal representations. Attention mechanisms were incorporated to focus the model on critical time points, with three variants: (1) custom attention, using a weight matrix with tanh activation and softmax normalization, (2) multi-head attention, inspired by transformer architectures, employing 1-4 attention heads with key dimensions ranging from 16 to 64, and (3) self-attention, implemented using TensorFlow’s native attention mechanism. The model also included a dense hidden layer (16–64 units), ReLU activation, and L2 regularization (0.0–0.1), followed by a final sigmoid output for binary classification. Hyperparameter tuning was carried out using Keras Tuner with 20 random search trials across LSTM units, dropout rates, attention mechanisms, dense layer configurations, and learning rates (0.001, 0.0005, 0.0001). Class weights were computed using scikit-learn’s compute_class_weight to handle class imbalance in elevated BP prediction. To leverage the complementary strengths of both models, we developed an ensemble strategy using weighted averaging. The final ensemble prediction was computed as:

5 $${\rm{Ensemble}}\,{\rm{prediction}}={\rm{\alpha }}\times {\rm{XGBoost}}\,{\rm{probability}}+{\rm{\beta }}\times {\rm{LSTM}}\,{\rm{probability}}$$

where the weights α and β were determined through an exhaustive grid search from 0.0 to 1.0 in increments of 0.1, with the optimal weights selected based on the validation set AUROC. This approach maximized discriminative ability by combining the feature engineering strengths of XGBoost with the temporal modeling capabilities of the LSTM.

### Evaluation methodology

Model performance was primarily evaluated using the Area Under the Receiver Operating Characteristic Curve (AUROC), which provides a threshold-independent measure of discriminative ability. We also calculated sensitivity and specificity at an “optimal” (i.e., balanced) threshold, determined by maximizing Youden’s index (sensitivity + specificity - 1). To quantify prediction uncertainty and ensure robust performance estimates, bootstrap analysis with 1000 iterations was employed. For each iteration, we resampled predictions with replacement and computed all evaluation metrics, enabling us to report 95% confidence intervals. We also performed threshold analysis across 101 evenly spaced values (0.00 to 1.00), computing sensitivity and specificity at each threshold to assess the full performance spectrum.

Model interpretability was achieved through SHAP (SHapley Additive exPlanations) analysis for feature attribution in the XGBoost pipeline and attention weight analysis, to understand the model’s focus on specific temporal points, for the LSTM model. Additionally, latent space analysis was performed using UMAP and t-SNE projections of learned representations, providing insights into how the models separated elevated BP events from normal physiological states.

### Grouped feature ablation

For each participant, we conducted a grouped feature ablation to assess the importance of individual feature families. We retrained the personalized model end-to-end while systematically removing one feature family at a time: heart rate statistics, step statistics, EMA-stress summaries, simple ratios, short cumulative windows, and calendar/time context. All other model settings were kept fixed, including preprocessing, train/validation splits, resampling choices, class weights, and hyperparameters, using the same random seed. No hyperparameter tuning was performed during the ablation process. Thresholds for classification were selected by maximizing Youden’s J on the participant’s validation data (across 101 threshold values from 0.00 to 1.00). To estimate prediction uncertainty, we conducted row-wise bootstrap resampling (1000 iterations) on the validation set, reporting 95% confidence intervals for AUROC and other operating metrics as applicable. Runs yielding AUROC = 0 or NaN were excluded from analysis and flagged as invalid in the figures and tables. In alignment with the study’s focus on individual-level analysis, we prioritize reporting on a per-participant basis. Cross-participant summaries, such as the frequency of critical feature families, are presented to provide context. Aggregate statistics, including means and 95% confidence intervals, are provided in the Supplementary material.

## Supplementary information

Supplementary information

## Data Availability

A small, de-identified subset of the dataset is publicly available in the project repository (https://github.com/ucsfdigitalhealth/CardioMate_ML_NOD) to support reproducibility of the analysis pipelines on representative data. This subset includes aligned Fitbit heart rate and step data, EMA stress responses, and BP measurements sufficient to replicate preprocessing and modeling workflows. The full dataset (40 participants across two studies; approximately 4 weeks per participant) will be described and released in a companion dataset paper currently in preparation.

## Code availability

All code used for data preprocessing, feature engineering, and model development is available at https://github.com/ucsfdigitalhealth/CardioMate_ML_NOD.

## References

[1] Pinge, A., Gad, V., Jaisighani, D., Ghosh, S. & Sen, S. Detection and monitoring of stress using wearables: a systematic review. *Front. Comput. Sci.* **6**, 1478851 (2024).

[2] Tomitani, N., Kanegae, H., Suzuki, Y., Kuwabara, M. & Kario, K. Stress-induced blood pressure elevation self-measured by a wearable watch-type device. *Am. J. Hypertension* **34**, 377–382 (2021). 10.1093/ajh/hpaa139 PMC8057129 32852527

[3] Vancheri, F., Longo, G., Vancheri, E. & Henein, M. Y. Mental Stress and Cardiovascular Health-Part I. *J. Clin. Med.* **11**, 3353 (2022). **10**. 35743423 10.3390/jcm11123353 PMC9225328

[4] Sabry, F., Eltaras, T., Labda, W., Alzoubi, K. & Malluhi, Q. Machine Learning for Healthcare Wearable Devices: The Big Picture. *J. Health Eng.* **2022**, 4653923 (2022). 10.1155/2022/4653923 PMC9038375 35480146

[5] Stone, A. A., Schneider, S. & Smyth, J. M. Evaluation of Pressing Issues in Ecological Momentary Assessment. *Annu Rev. Clin. Psychol.* **19**, 107–131 (2023). 36475718 10.1146/annurev-clinpsy-080921-083128 PMC12991416

[6] Li, J. & Washington, P. A comparison of personalized and generalized approaches to emotion detection using wearable biosignals: machine learning study. *JMIR AI* **3**, e52171 (2024). 38875573 10.2196/52171 PMC11127131

[7] Ninh, M. et al. An improved subject-independent stress detection model applied to consumer-grade wearable devices through hybrid sampling and EfficientNetB0. in *Proc. of the 28th International Conference on Multimedia Modeling* (*MMM*, 2022).

[8] Kargarandehkordi, A., Kaisti, M. & Washington, P. Personalization of affective models using classical machine learning: a feasibility study. *Appl. Sci.* **14**, 1337 (2024). 40771772 10.3390/app14041337 PMC12327430

[9] Shah, R. V. et al. Personalized machine learning of depressed mood using wearables. *Transl. Psychiatry* **11**, 338 (2021). 34103481 10.1038/s41398-021-01445-0 PMC8187630

[10] Olyanasab, A. & Alihosseini, M. A. Leveraging machine learning for personalized wearable biomedical devices: a review. *J. Pers. Med.* **14**, 203 (2024). 38392636 10.3390/jpm14020203 PMC10890129

[11] Cvach, M. Monitor alarm fatigue: an integrative review. *Biomed. Instrum. Technol.* **46**, 268–277 (2012). 22839984 10.2345/0899-8205-46.4.268

[12] Nahum-Shani, I. et al. Just-in-Time Adaptive Interventions (JITAIs) in Mobile Health: Key Components and Design Principles for Ongoing Health Behavior Support. *Ann. Behav. Med.* **52**, 446–462 (2018). 27663578 10.1007/s12160-016-9830-8 PMC5364076

[13] Sendelbach, S. & Funk, M. Alarm Fatigue: A Patient Safety Concern. *AACN Adv. Crit. Care* **24**, 378–386 (2013). 24153215 10.1097/NCI.0b013e3182a903f9

[14] Kargarandehkordi, A., Slade, C. & Washington, P. Personalized AI-Driven Real-Time Models to Predict Stress-Induced Blood Pressure Spikes Using Wearable Devices: Proposal for a Prospective Cohort Study. *JMIR Res. Protoc.* **13**, 55615 (2024). 10.2196/55615 PMC11002732 38526539

[15] Sun, Y. et al. Personalized deep learning for substance use in Hawaii: protocol for a passive sensing and ecological momentary assessment study. *JMIR Res. Protoc.* **13**, e46493 (2024). 38324375 10.2196/46493 PMC10882478

[16] Klimek, A., Mannheim, I., Schouten, G., Wouters, E. J. M. & Peeters, M. W. H. Wearables measuring electrodermal activity to assess perceived stress in care: a scoping review. *Acta Neuropsychiatrica* **37**, e19 (2025). 10.1017/neu.2023.19 PMC13130278 36960675

[17] Kario, K., Harada, N. & Okura, A. Digital therapeutics in hypertension: evidence and perspectives. *Hypertension* **79**, 2148–2158 (2022). 35726619 10.1161/HYPERTENSIONAHA.122.19414 PMC9444254

[18] ML, G. R. et al. Wearables for Stress Management: A Scoping Review. *Healthc. (Basel)* **11**, 2369 (2023). 10.3390/healthcare11172369 PMC10486660 37685403

[19] Sirbu, V. & David, O. A. Efficacy of app-based mobile health interventions for stress management: A systematic review and meta-analysis of self-reported, physiological, and neuroendocrine stress-related outcomes. *Clin. Psychol. Rev.* **114**, 102515 (2024). 39522422 10.1016/j.cpr.2024.102515

[20] Li, S. et al. Monitoring Substance Use with Fitbit Biosignals: A Case Study on Training Deep Learning Models Using Ecological Momentary Assessments and Passive Sensing. *AI* **5**, 2725–2738 (2024). 40351335 10.3390/ai5040131 PMC12065672

[21] Ronca, V. et al. Wearable technologies for electrodermal and cardiac activity measurements: a comparison between fitbit sense, empatica E4 and shimmer GSR3+. *Sensors* **23**, 5847 (2023). 37447697 10.3390/s23135847 PMC10346781

[22] Bent, B., Goldstein, B. A., Kibbe, W. A. & Dunn, J. P. Investigating sources of inaccuracy in wearable optical heart rate sensors. *NPJ Digital Med.* **3**, 18 (2020). 10.1038/s41746-020-0226-6 PMC7010823 32047863

