# Supplementary material for: Personalized modeling of stress and blood pressure reactivity using mobile health data
Source: Npj Ment Health Res. 2026 May 8;5:37. doi: 10.1038/s44184-026-00210-9 (PMC13396679; doi:10.1038/s44184-026-00210-9)
Supplement: Supplementary file 1 — Supplementary information [file 44184_2026_210_MOESM1_ESM.pdf]

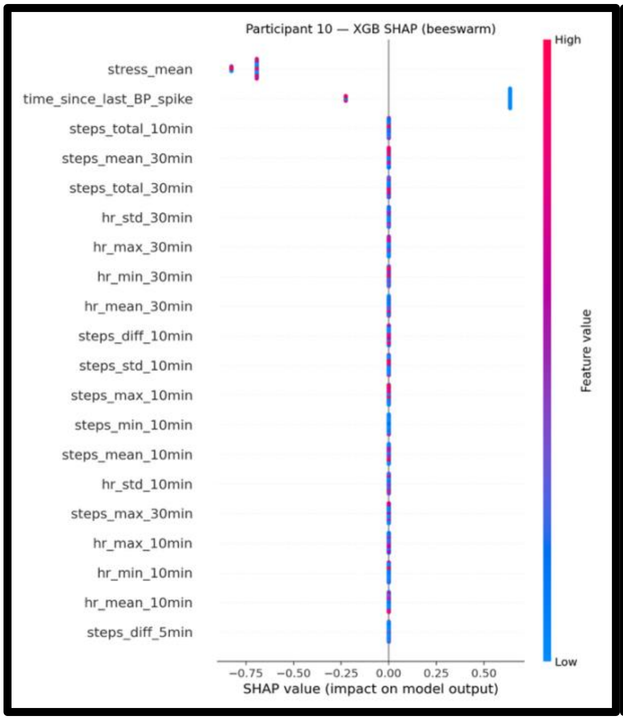

Participant 10 — XGB SHAP (beeswarm)
stress_mean
time_since_last_BP_spike
steps_total_10min
steps_mean_30min
steps_total_30min
hr_std_30min
hr_max_30min
hr_min_30min
hr_mean_30min
steps_diff_10min
steps_std_10min
steps_max_10min
steps_min_10min
steps_mean_10min
hr_std_10min
steps_max_30min
hr_max_10min
hr_min_10min
hr_mean_10min
steps_diff_5min
−0.75 −0.50 −0.25 0.00 0.25 0.50
SHAP value (impact on model output)
High
Feature value
Low

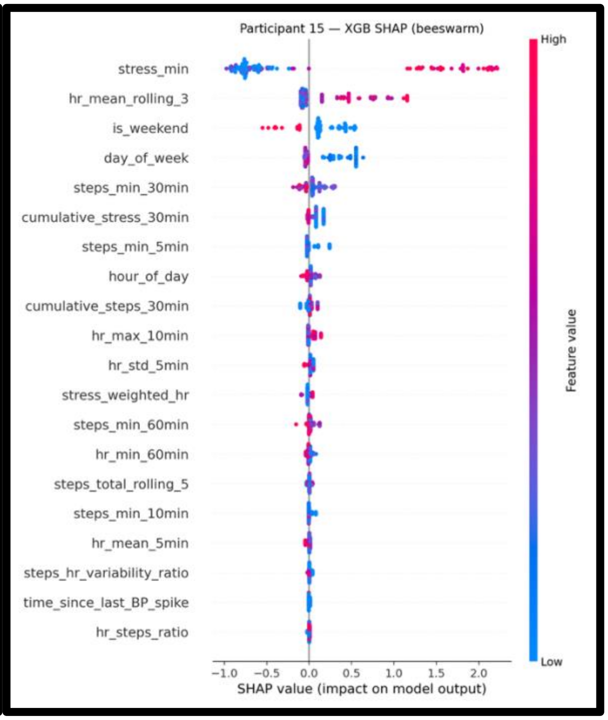

Participant 15 — XGB SHAP (beeswarm)
stress_min
hr_mean_rolling_3
is_weekend
day_of_week
steps_min_30min
cumulative_stress_30min
steps_min_5min
hour_of_day
cumulative_steps_30min
hr_max_10min
hr_std_5min
stress_weighted_hr
steps_min_60min
hr_min_60min
steps_total_rolling_5
steps_min_10min
hr_mean_5min
steps_hr_variability_ratio
time_since_last_BP_spike
hr_steps_ratio
−1.0 −0.5 0.0 0.5 1.0 1.5 2.0
SHAP value (impact on model output)
High
Feature value
Low

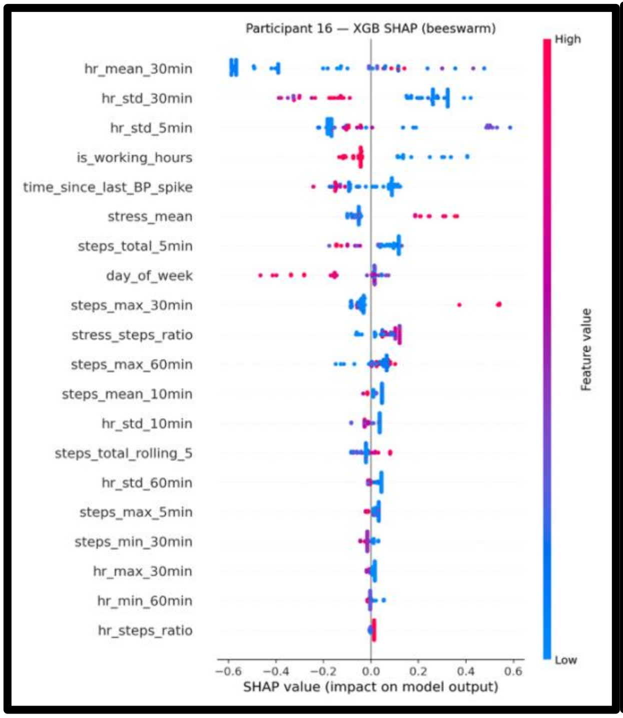

Participant 16 — XGB SHAP (beeswarm)
hr_mean_30min
hr_std_30min
hr_std_5min
is_working_hours
time_since_last_BP_spike
stress_mean
steps_total_5min
day_of_week
steps_max_30min
stress_steps_ratio
steps_max_60min
steps_mean_10min
hr_std_10min
steps_total_rolling_5
hr_std_60min
steps_max_5min
steps_min_30min
hr_max_30min
hr_min_60min
hr_steps_ratio
−0.6 −0.4 −0.2 0.0 0.2 0.4 0.6
SHAP value (impact on model output)
High
Feature value
Low

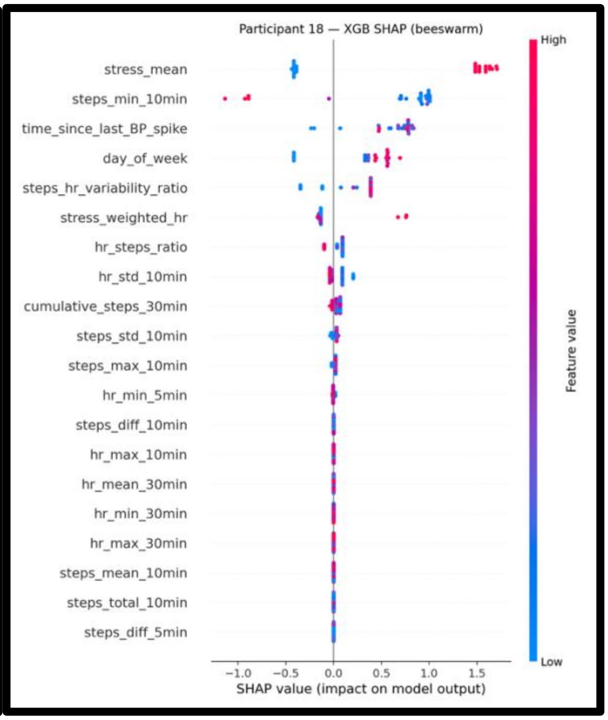

Participant 18 — XGB SHAP (beeswarm)
stress_mean
steps_min_10min
time_since_last_BP_spike
day_of_week
steps_hr_variability_ratio
stress_weighted_hr
hr_steps_ratio
hr_std_10min
cumulative_steps_30min
steps_std_10min
steps_max_10min
hr_min_5min
steps_diff_10min
hr_max_10min
hr_mean_30min
hr_min_30min
hr_max_30min
steps_mean_10min
steps_total_10min
steps_diff_5min
−1.0 −0.5 0.0 0.5 1.0 1.5
SHAP value (impact on model output)
High
Feature value
Low

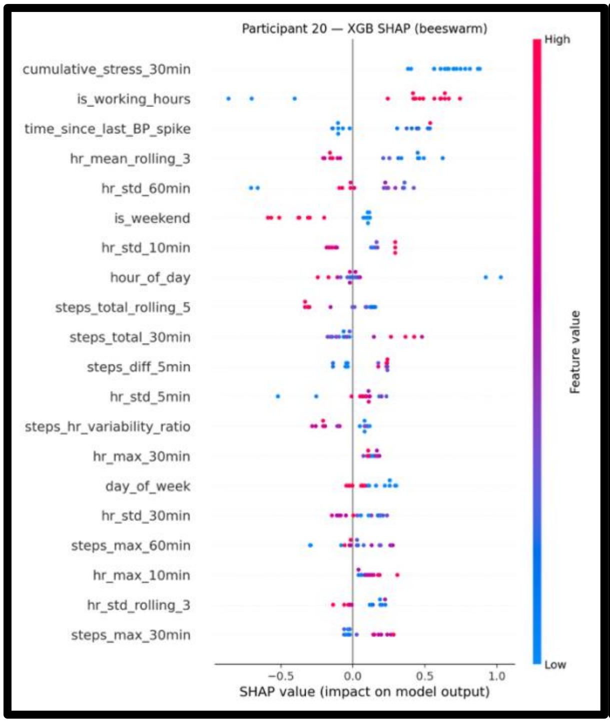
Participant 20 — XGB SHAP (beeswarm)
cumulative_stress_30min
is_working_hours
time_since_last_BP_spike
hr_mean_rolling_3
hr_std_60min
is_weekend
hr_std_10min
hour_of_day
steps_total_rolling_5
steps_total_30min
steps_diff_5min
hr_std_5min
steps_hr_variability_ratio
hr_max_30min
day_of_week
hr_std_30min
steps_max_60min
hr_max_10min
hr_std_rolling_3
steps_max_30min
−0.5
0.0
0.5
1.0
SHAP value (impact on model output)
High
Feature value
Low

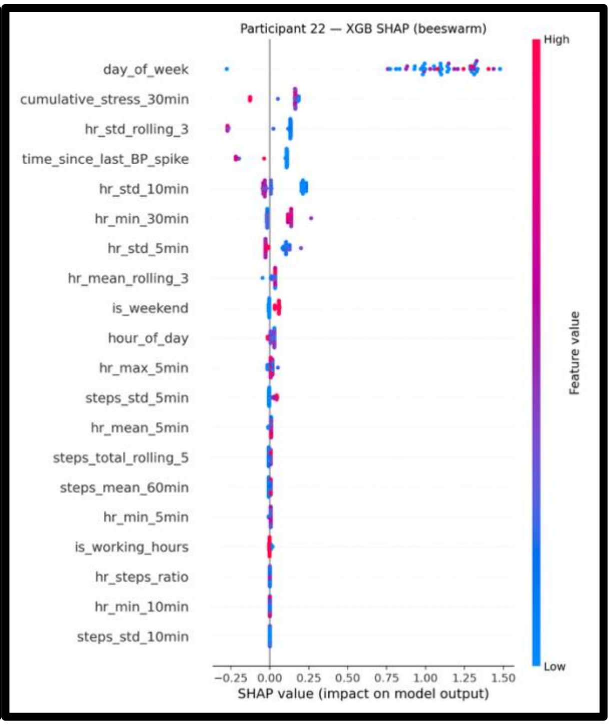
Participant 22 — XGB SHAP (beeswarm)
day_of_week
cumulative_stress_30min
hr_std_rolling_3
time_since_last_BP_spike
hr_std_10min
hr_min_30min
hr_std_5min
hr_mean_rolling_3
is_weekend
hour_of_day
hr_max_5min
steps_std_5min
hr_mean_5min
steps_total_rolling_5
steps_mean_60min
hr_min_5min
is_working_hours
hr_steps_ratio
hr_min_10min
steps_std_10min
−0.25
0.00
0.25
0.50
0.75
1.00
1.25
1.50
SHAP value (impact on model output)
High
Feature value
Low

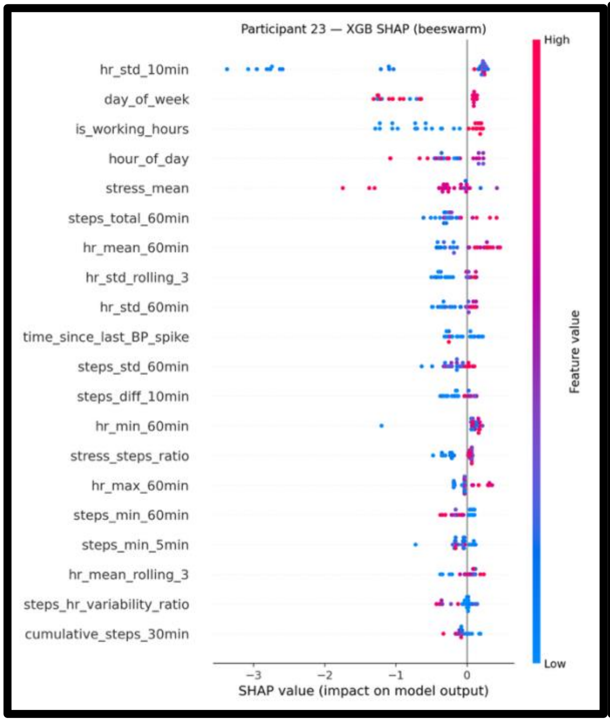
Participant 23 — XGB SHAP (beeswarm)
hr_std_10min
day_of_week
is_working_hours
hour_of_day
stress_mean
steps_total_60min
hr_mean_60min
hr_std_rolling_3
hr_std_60min
time_since_last_BP_spike
steps_std_60min
steps_diff_10min
hr_min_60min
stress_steps_ratio
hr_max_60min
steps_min_60min
steps_min_5min
hr_mean_rolling_3
steps_hr_variability_ratio
cumulative_steps_30min
−3
−2
−1
0
SHAP value (impact on model output)
High
Feature value
Low

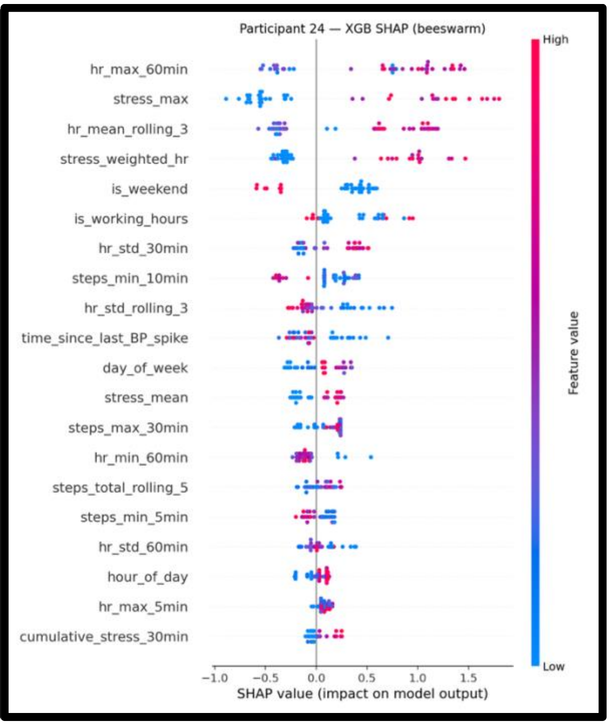
Participant 24 — XGB SHAP (beeswarm)
hr_max_60min
stress_max
hr_mean_rolling_3
stress_weighted_hr
is_weekend
is_working_hours
hr_std_30min
steps_min_10min
hr_std_rolling_3
time_since_last_BP_spike
day_of_week
stress_mean
steps_max_30min
hr_min_60min
steps_total_rolling_5
steps_min_5min
hr_std_60min
hour_of_day
hr_max_5min
cumulative_stress_30min
−1.0
−0.5
0.0
0.5
1.0
1.5
SHAP value (impact on model output)
High
Feature value
Low

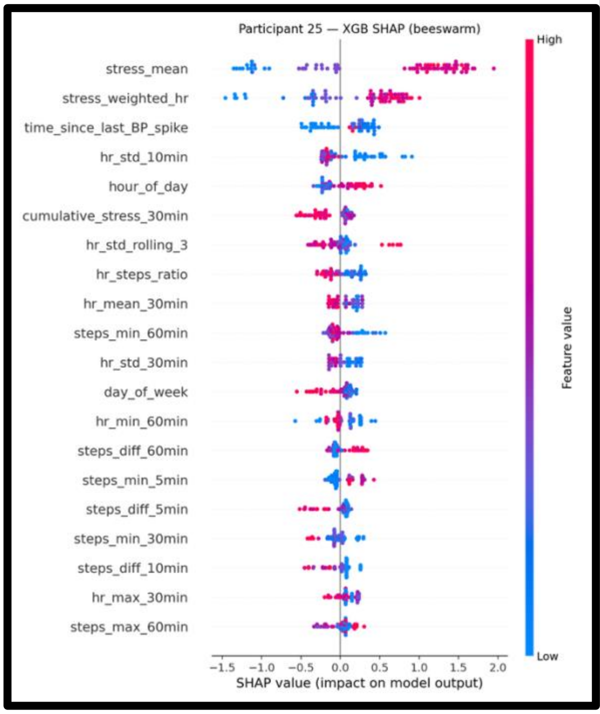
Participant 25 — XGB SHAP (beeswarm)
stress_mean
stress_weighted_hr
time_since_last_BP_spike
hr_std_10min
hour_of_day
cumulative_stress_30min
hr_std_rolling_3
hr_steps_ratio
hr_mean_30min
steps_min_60min
hr_std_30min
day_of_week
hr_min_60min
steps_diff_60min
steps_min_5min
steps_diff_5min
steps_min_30min
steps_diff_10min
hr_max_30min
steps_max_60min
−1.5 −1.0 −0.5 0.0 0.5 1.0 1.5 2.0
SHAP value (impact on model output)
High
Feature value
Low

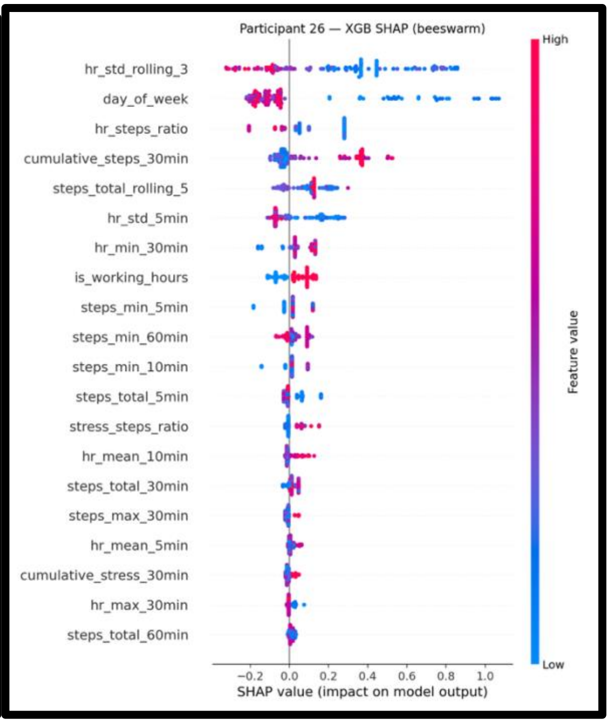
Participant 26 — XGB SHAP (beeswarm)
hr_std_rolling_3
day_of_week
hr_steps_ratio
cumulative_steps_30min
steps_total_rolling_5
hr_std_5min
hr_min_30min
is_working_hours
steps_min_5min
steps_min_60min
steps_min_10min
steps_total_5min
stress_steps_ratio
hr_mean_10min
steps_total_30min
steps_max_30min
hr_mean_5min
cumulative_stress_30min
hr_max_30min
steps_total_60min
−0.2 0.0 0.2 0.4 0.6 0.8 1.0
SHAP value (impact on model output)
High
Feature value
Low

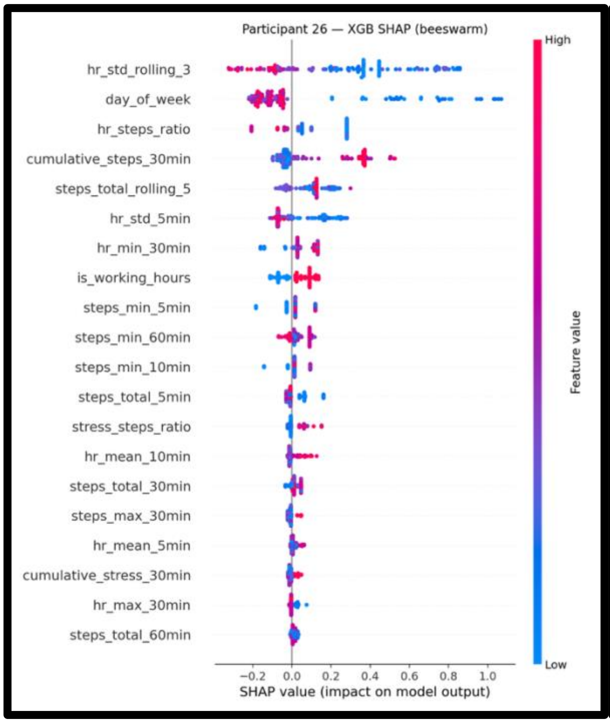
Participant 26 — XGB SHAP (beeswarm)
hr_std_rolling_3
day_of_week
hr_steps_ratio
cumulative_steps_30min
steps_total_rolling_5
hr_std_5min
hr_min_30min
is_working_hours
steps_min_5min
steps_min_60min
steps_min_10min
steps_total_5min
stress_steps_ratio
hr_mean_10min
steps_total_30min
steps_max_30min
hr_mean_5min
cumulative_stress_30min
hr_max_30min
steps_total_60min
−0.2 0.0 0.2 0.4 0.6 0.8 1.0
SHAP value (impact on model output)
High
Feature value
Low

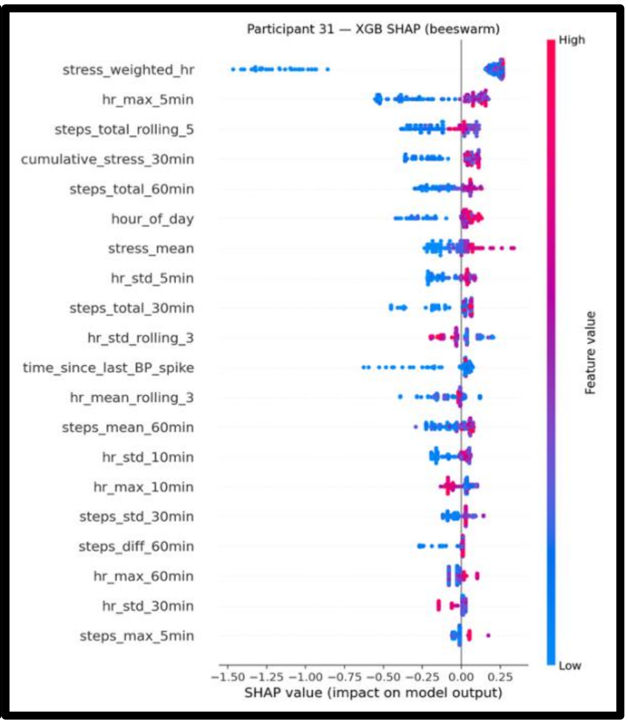
Participant 31 — XGB SHAP (beeswarm)
stress_weighted_hr
hr_max_5min
steps_total_rolling_5
cumulative_stress_30min
steps_total_60min
hour_of_day
stress_mean
hr_std_5min
steps_total_30min
hr_std_rolling_3
time_since_last_BP_spike
hr_mean_rolling_3
steps_mean_60min
hr_std_10min
hr_max_10min
steps_std_30min
steps_diff_60min
hr_max_60min
hr_std_30min
steps_max_5min
−1.50 −1.25 −1.00 −0.75 −0.50 −0.25 0.00 0.25
SHAP value (impact on model output)
High
Feature value
Low

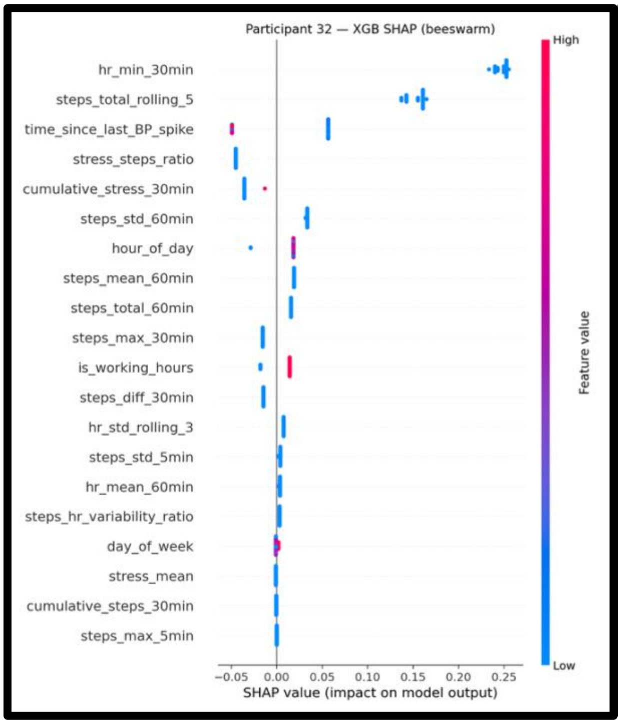
Participant 32 — XGB SHAP (beeswarm)
hr_min_30min
steps_total_rolling_5
time_since_last_BP_spike
stress_steps_ratio
cumulative_stress_30min
steps_std_60min
hour_of_day
steps_mean_60min
steps_total_60min
steps_max_30min
is_working_hours
steps_diff_30min
hr_std_rolling_3
steps_std_5min
hr_mean_60min
steps_hr_variability_ratio
day_of_week
stress_mean
cumulative_steps_30min
steps_max_5min
−0.05 0.00 0.05 0.10 0.15 0.20 0.25
SHAP value (impact on model output)
High
Feature value
Low

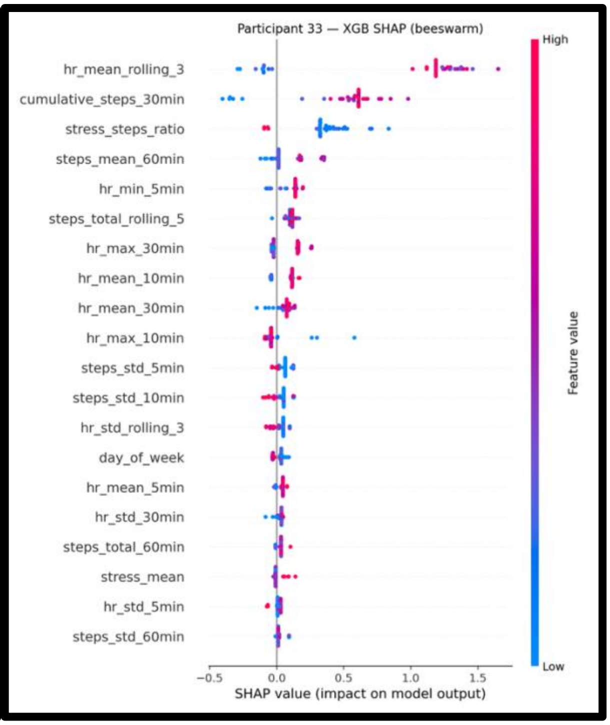
Participant 33 — XGB SHAP (beeswarm)
hr_mean_rolling_3
cumulative_steps_30min
stress_steps_ratio
steps_mean_60min
hr_min_5min
steps_total_rolling_5
hr_max_30min
hr_mean_10min
hr_mean_30min
hr_max_10min
steps_std_5min
steps_std_10min
hr_std_rolling_3
day_of_week
hr_mean_5min
hr_std_30min
steps_total_60min
stress_mean
hr_std_5min
steps_std_60min
−0.5 0.0 0.5 1.0 1.5
SHAP value (impact on model output)
High
Feature value
Low

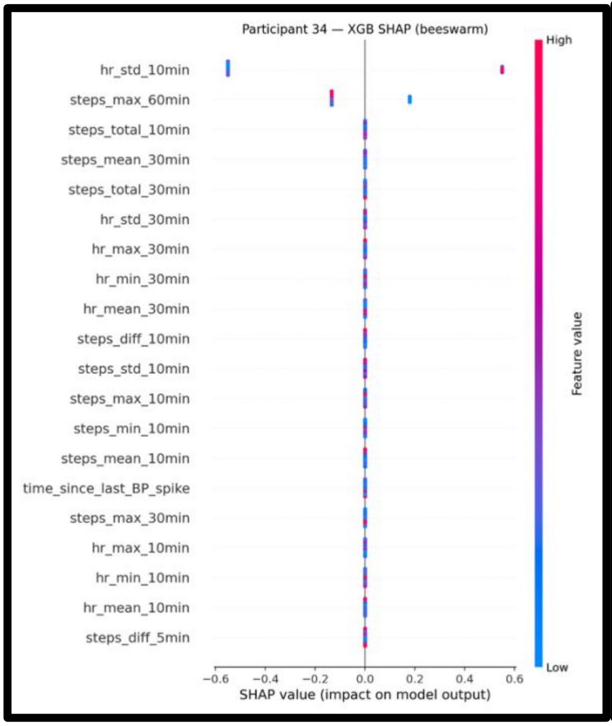
Participant 34 — XGB SHAP (beeswarm)
hr_std_10min
steps_max_60min
steps_total_10min
steps_mean_30min
steps_total_30min
hr_std_30min
hr_max_30min
hr_min_30min
hr_mean_30min
steps_diff_10min
steps_std_10min
steps_max_10min
steps_min_10min
steps_mean_10min
time_since_last_BP_spike
steps_max_30min
hr_max_10min
hr_min_10min
hr_mean_10min
steps_diff_5min
−0.6 −0.4 −0.2 0.0 0.2 0.4 0.6
SHAP value (impact on model output)
High
Feature value
Low

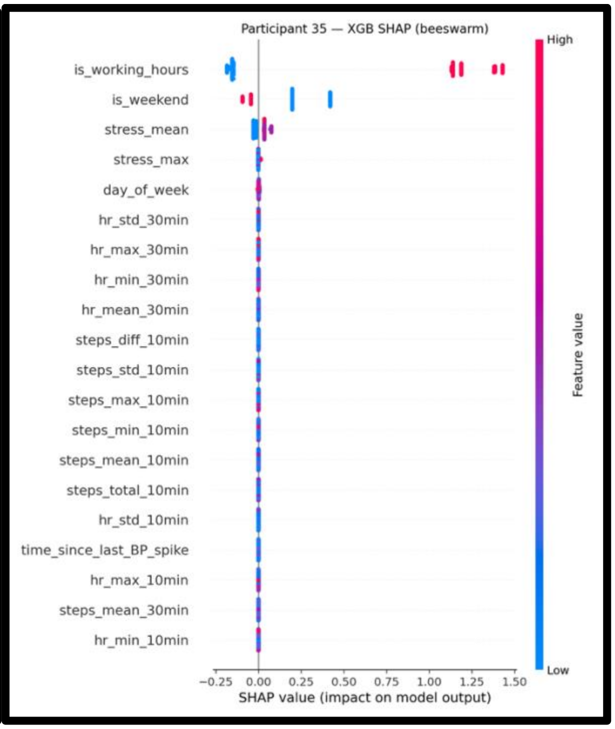
Participant 35 — XGB SHAP (beeswarm)
is_working_hours
is_weekend
stress_mean
stress_max
day_of_week
hr_std_30min
hr_max_30min
hr_min_30min
hr_mean_30min
steps_diff_10min
steps_std_10min
steps_max_10min
steps_min_10min
steps_mean_10min
steps_total_10min
hr_std_10min
time_since_last_BP_spike
hr_max_10min
steps_mean_30min
hr_min_10min
−0.25 0.00 0.25 0.50 0.75 1.00 1.25 1.50
SHAP value (impact on model output)
High
Feature value
Low

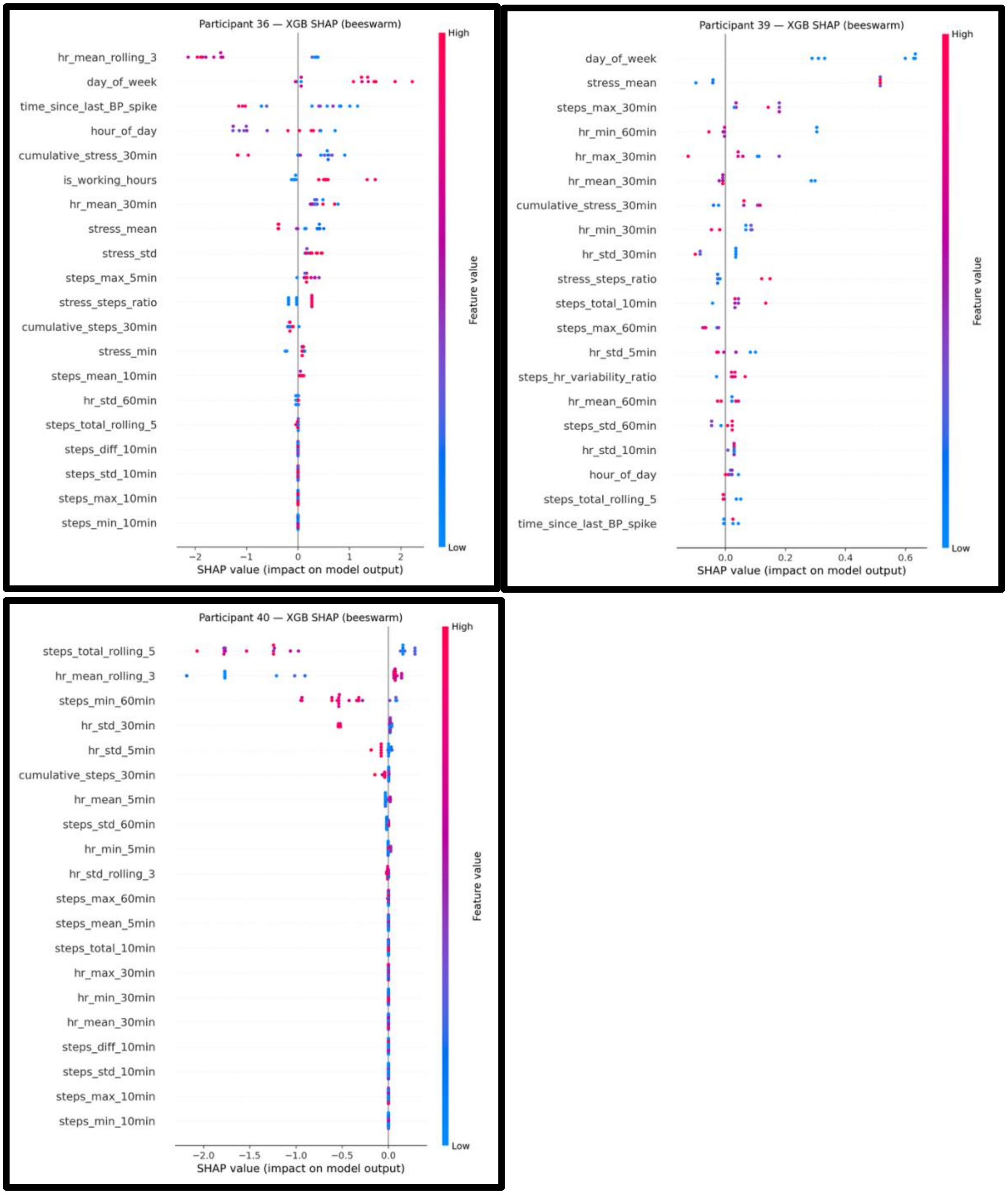

Figure 1. Per-participant feature attributions (SHAP) for personalised models. Twenty SHAP summary plots, one per participant; IDs increase row-wise from the top-left to the bottom-right. Within each panel, features are ordered by mean absolute SHAP value; each point represents one held-out test instance with horizontal position indicating the feature's marginal contribution to the log-odds of an elevated BP, and colour encoding the feature value (blue = low, pink = high). SHAP values were computed with TreeExplainer on the participant-specific XGBoost classifier (after scaling); feature values shown are unscaled for interpretability. Recurrent high-impact features across

participants include short-horizon HR/steps statistics, while rankings vary by individual, underscoring the value of personalised modelling.

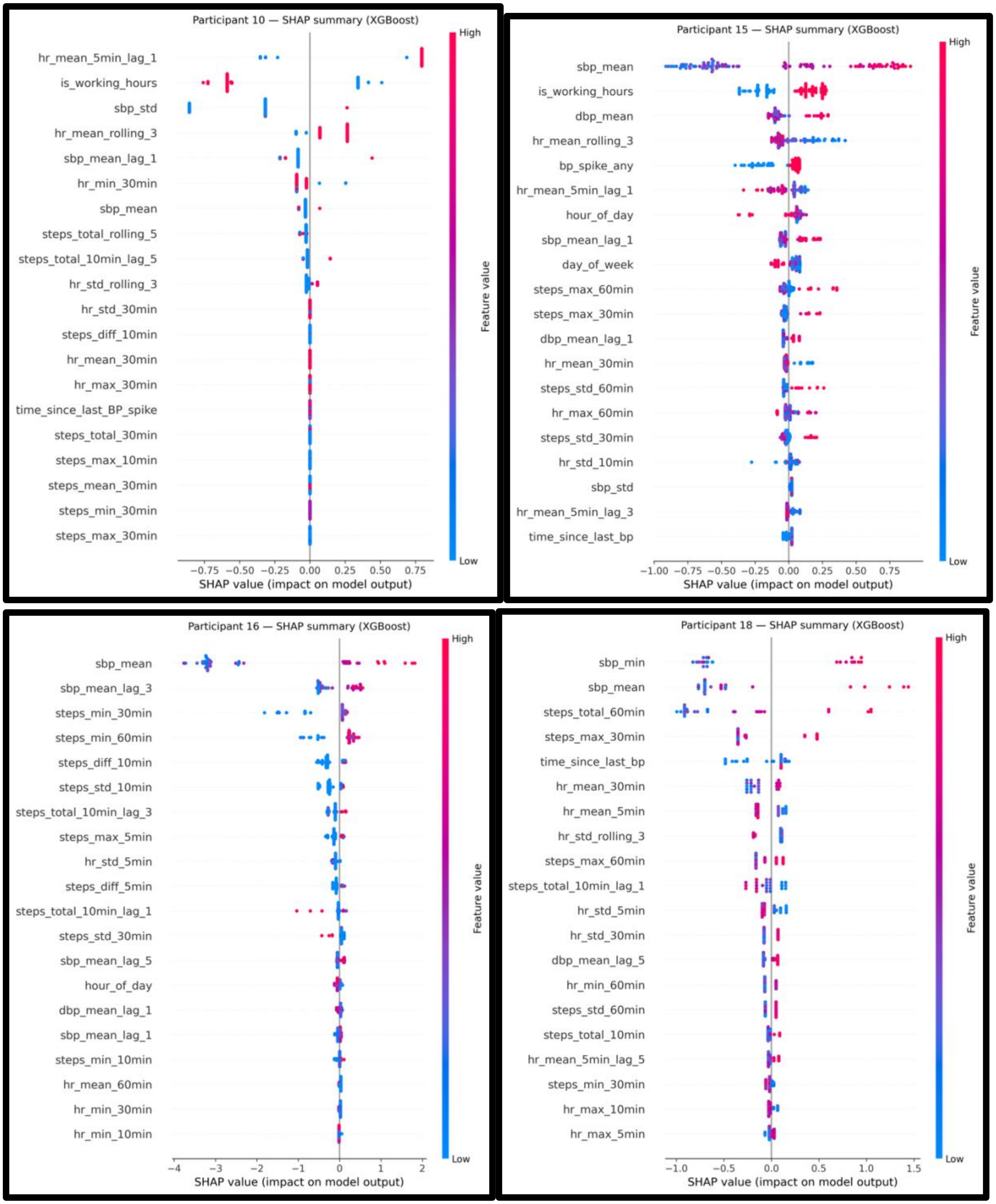

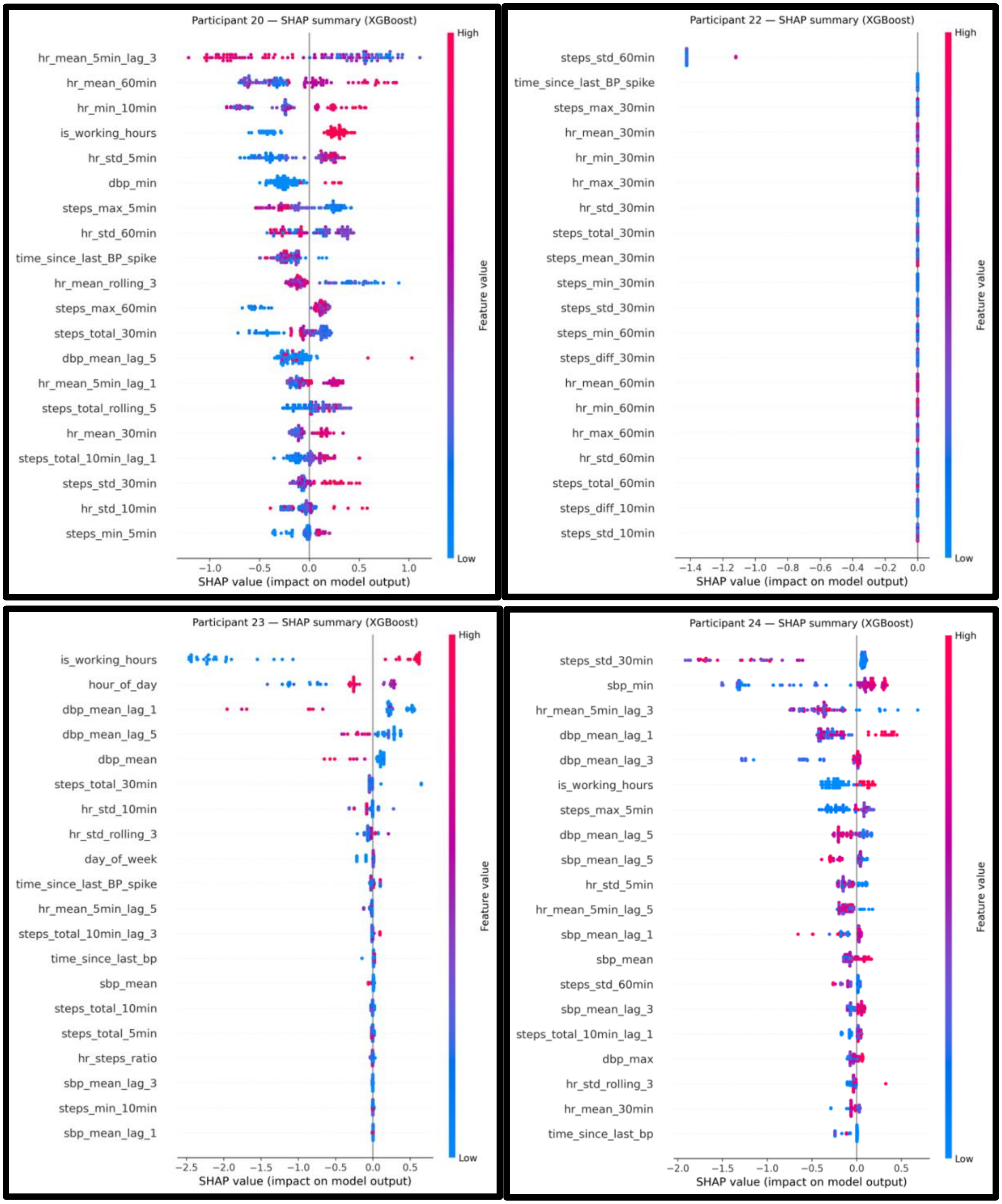

Participant 20 — SHAP summary (XGBoost)
hr_mean_5min_lag_3
hr_mean_60min
hr_min_10min
is_working_hours
hr_std_5min
dbp_min
steps_max_5min
hr_std_60min
time_since_last_BP_spike
hr_mean_rolling_3
steps_max_60min
steps_total_30min
dbp_mean_lag_5
hr_mean_5min_lag_1
steps_total_rolling_5
hr_mean_30min
steps_total_10min_lag_1
steps_std_30min
hr_std_10min
steps_min_5min
−1.0 −0.5 0.0 0.5 1.0
SHAP value (impact on model output)
High
Feature value
Low
Participant 22 — SHAP summary (XGBoost)
steps_std_60min
time_since_last_BP_spike
steps_max_30min
hr_mean_30min
hr_min_30min
hr_max_30min
hr_std_30min
steps_total_30min
steps_mean_30min
steps_min_30min
steps_std_30min
steps_min_60min
steps_diff_30min
hr_mean_60min
hr_min_60min
hr_max_60min
hr_std_60min
steps_total_60min
steps_diff_10min
steps_std_10min
−1.4 −1.2 −1.0 −0.8 −0.6 −0.4 −0.2 0.0
SHAP value (impact on model output)
High
Feature value
Low
Participant 23 — SHAP summary (XGBoost)
is_working_hours
hour_of_day
dbp_mean_lag_1
dbp_mean_lag_5
dbp_mean
steps_total_30min
hr_std_10min
hr_std_rolling_3
day_of_week
time_since_last_BP_spike
hr_mean_5min_lag_5
steps_total_10min_lag_3
time_since_last_bp
sbp_mean
steps_total_10min
steps_total_5min
hr_steps_ratio
sbp_mean_lag_3
steps_min_10min
sbp_mean_lag_1
−2.5 −2.0 −1.5 −1.0 −0.5 0.0 0.5
SHAP value (impact on model output)
High
Feature value
Low
Participant 24 — SHAP summary (XGBoost)
steps_std_30min
sbp_min
hr_mean_5min_lag_3
dbp_mean_lag_1
dbp_mean_lag_3
is_working_hours
steps_max_5min
dbp_mean_lag_5
sbp_mean_lag_5
hr_std_5min
hr_mean_5min_lag_5
sbp_mean_lag_1
sbp_mean
steps_std_60min
sbp_mean_lag_3
steps_total_10min_lag_1
dbp_max
hr_std_rolling_3
hr_mean_30min
time_since_last_bp
−2.0 −1.5 −1.0 −0.5 0.0 0.5
SHAP value (impact on model output)
High
Feature value
Low

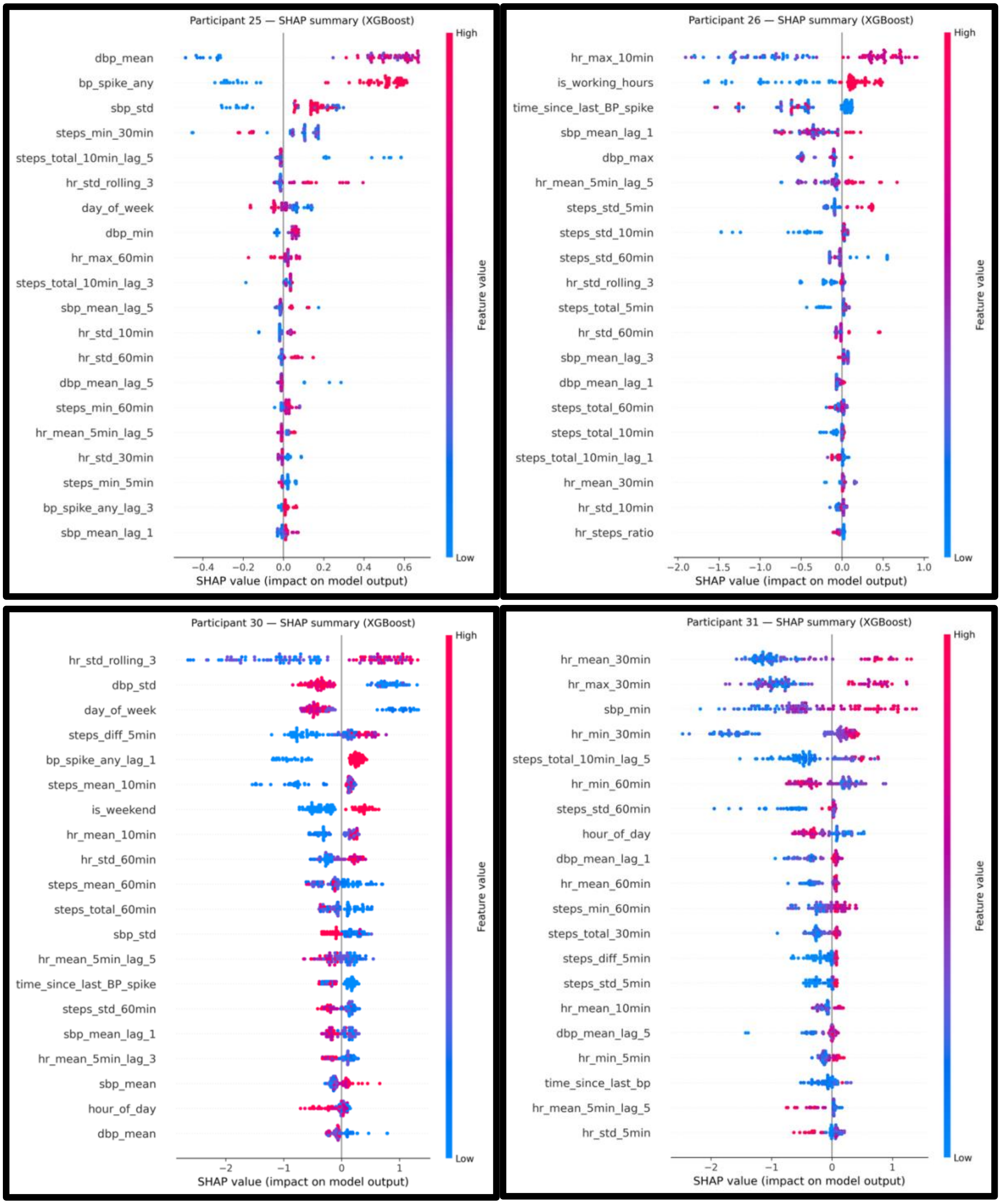

Participant 25 — SHAP summary (XGBoost)
dbp_mean
bp_spike_any
sbp_std
steps_min_30min
steps_total_10min_lag_5
hr_std_rolling_3
day_of_week
dbp_min
hr_max_60min
steps_total_10min_lag_3
sbp_mean_lag_5
hr_std_10min
hr_std_60min
dbp_mean_lag_5
steps_min_60min
hr_mean_5min_lag_5
hr_std_30min
steps_min_5min
bp_spike_any_lag_3
sbp_mean_lag_1
−0.4 −0.2 0.0 0.2 0.4 0.6
SHAP value (impact on model output)
High
Feature value
Low
Participant 26 — SHAP summary (XGBoost)
hr_max_10min
is_working_hours
time_since_last_BP_spike
sbp_mean_lag_1
dbp_max
hr_mean_5min_lag_5
steps_std_5min
steps_std_10min
steps_std_60min
hr_std_rolling_3
steps_total_5min
hr_std_60min
sbp_mean_lag_3
dbp_mean_lag_1
steps_total_60min
steps_total_10min
steps_total_10min_lag_1
hr_mean_30min
hr_std_10min
hr_steps_ratio
−2.0 −1.5 −1.0 −0.5 0.0 0.5 1.0
SHAP value (impact on model output)
High
Feature value
Low
Participant 30 — SHAP summary (XGBoost)
hr_std_rolling_3
dbp_std
day_of_week
steps_diff_5min
bp_spike_any_lag_1
steps_mean_10min
is_weekend
hr_mean_10min
hr_std_60min
steps_mean_60min
steps_total_60min
sbp_std
hr_mean_5min_lag_5
time_since_last_BP_spike
steps_std_60min
sbp_mean_lag_1
hr_mean_5min_lag_3
sbp_mean
hour_of_day
dbp_mean
−2 −1 0 1
SHAP value (impact on model output)
High
Feature value
Low
Participant 31 — SHAP summary (XGBoost)
hr_mean_30min
hr_max_30min
sbp_min
hr_min_30min
steps_total_10min_lag_5
hr_min_60min
steps_std_60min
hour_of_day
dbp_mean_lag_1
hr_mean_60min
steps_min_60min
steps_total_30min
steps_diff_5min
steps_std_5min
hr_mean_10min
dbp_mean_lag_5
hr_min_5min
time_since_last_bp
hr_mean_5min_lag_5
hr_std_5min
−2 −1 0 1
SHAP value (impact on model output)
High
Feature value
Low

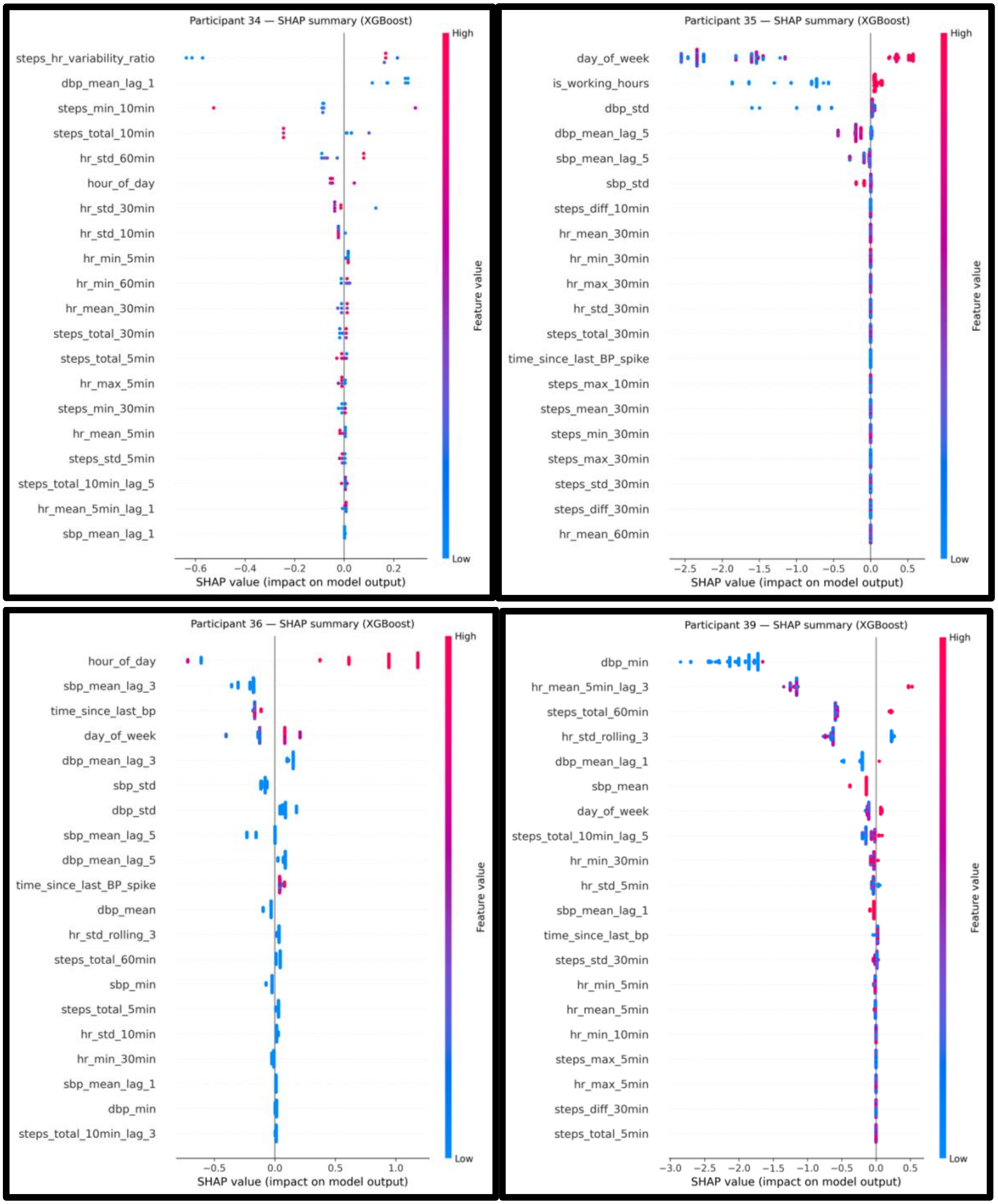

Figure 2. Per-participant SHAP summaries for the personalised "high-stress" models (IDs increasing row-wise). Participants 17, 32, 33, 40 were excluded due to insufficient high-stress labels.

Note 1: Temporal Stability of Model Performance

We evaluated the minimum monitoring duration required for stable feature reliability and predictive accuracy by retraining models from scratch for each duration (3-29 days) using a consistent 75:25 day-level train-test split to prevent data leakage.

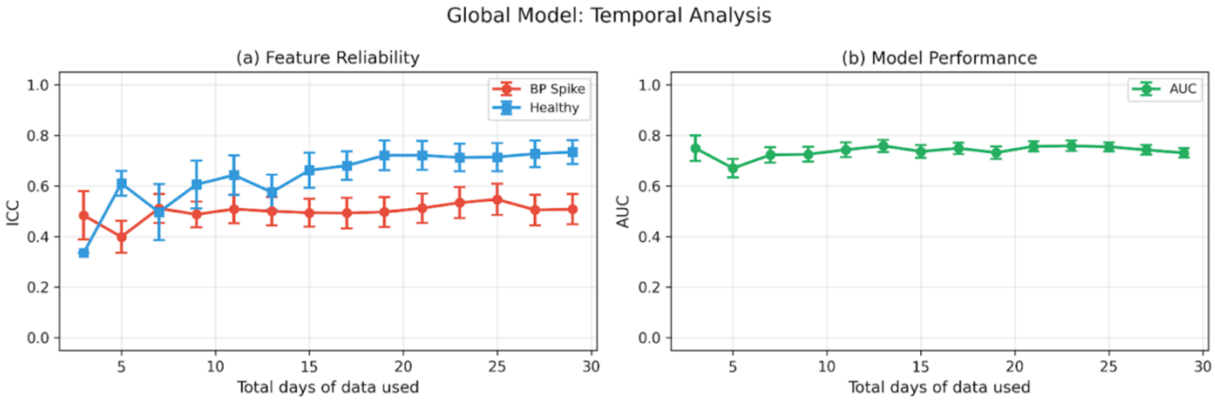

Figure 3. ICC Analysis of (a) Feature Reliability and (b) Model Performance of Global Model.

For the global model (Figure 3a), intraclass correlation coefficients (ICC) for elevated BP features showed moderate reliability, starting at 0.50 ± 0.08 at 3 days and stabilizing around 0.53 ± 0.04 from 15 days onward, indicating consistent but modest within-subject reliability for elevated-related physiological features. Healthy-state features demonstrated substantially higher reliability across all durations, beginning at 0.35 ± 0.08 at 3 days and reaching a plateau of approximately 0.75 ± 0.04 from 15-2830 days. This pronounced difference suggests that baseline physiological patterns during non-elevated periods exhibit greater temporal consistency and are more reliably measurable than the dynamic changes associated with elevated BP events.

Predictive accuracy of the global ensemble showed rapid initial improvement (Figure 3b), rising from AUROC = 0.75 ± 0.04 at 3 days to a stable plateau of 0.73-0.74 ± 0.02 from 7 days onward when tested on a fixed 7-day period. While performance stabilized quickly, it remained below the target threshold of 0.85, suggesting that the fixed test duration approach may underestimate model capabilities compared to variable-length evaluation periods. Bootstrap confidence intervals narrowed consistently with longer training durations (±0.04 at 3 days to ±0.02 at 23 days), indicating improved precision in performance estimates.

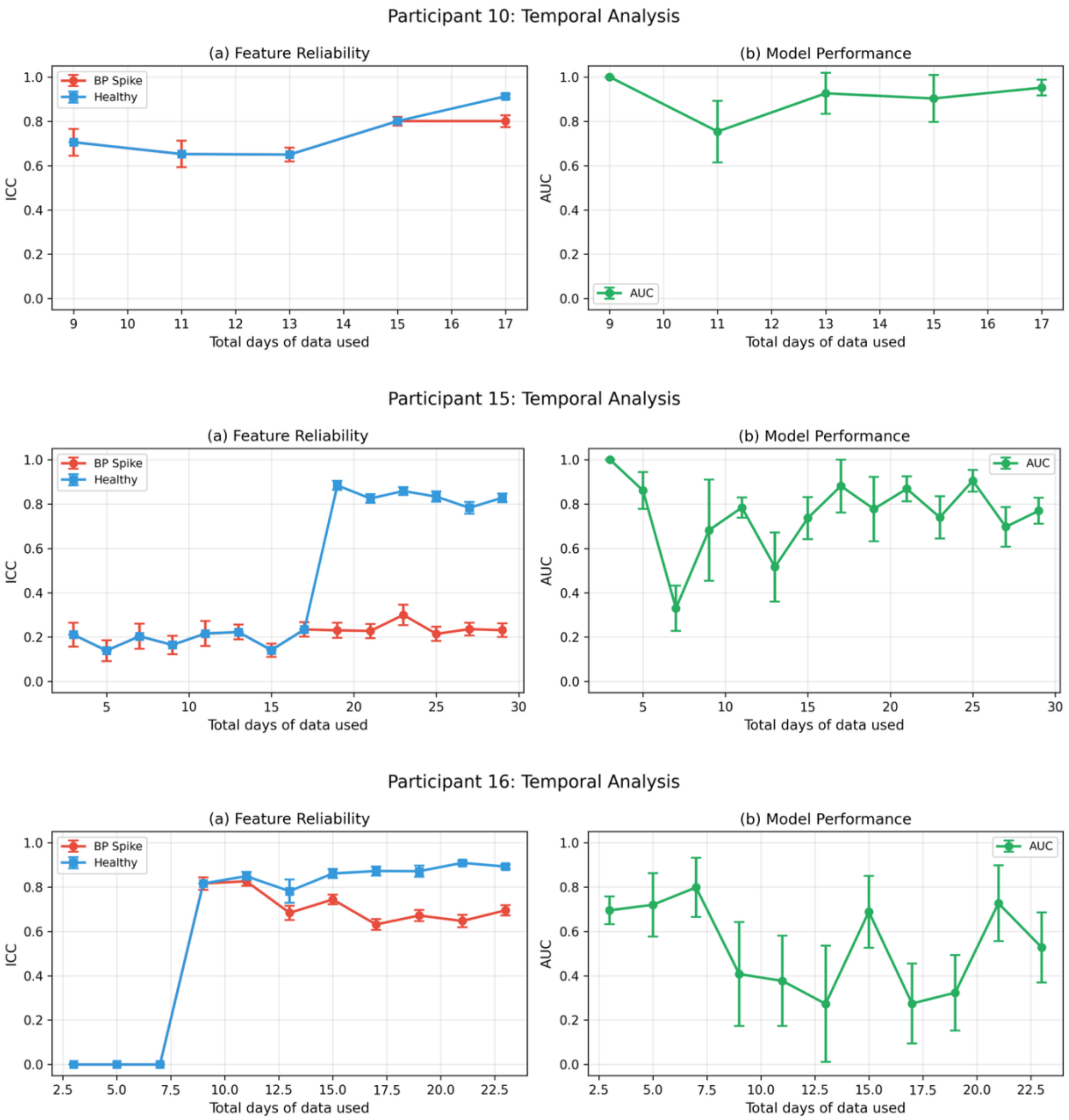
Participant 10: Temporal Analysis
(a) Feature Reliability
BP Spike
Healthy
ICC
Total days of data used
(b) Model Performance
AUC
Total days of data used
Participant 15: Temporal Analysis
(a) Feature Reliability
BP Spike
Healthy
ICC
Total days of data used
(b) Model Performance
AUC
Total days of data used
Participant 16: Temporal Analysis
(a) Feature Reliability
BP Spike
Healthy
ICC
Total days of data used
(b) Model Performance
AUC
Total days of data used

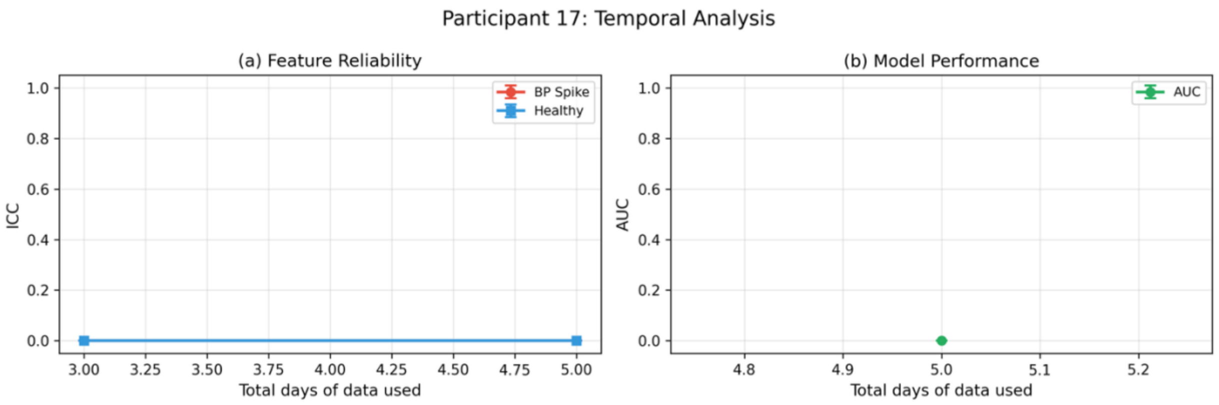
Participant 17: Temporal Analysis
(a) Feature Reliability
ICC
BP Spike
Healthy
Total days of data used
(b) Model Performance
AUC
AUC
Total days of data used

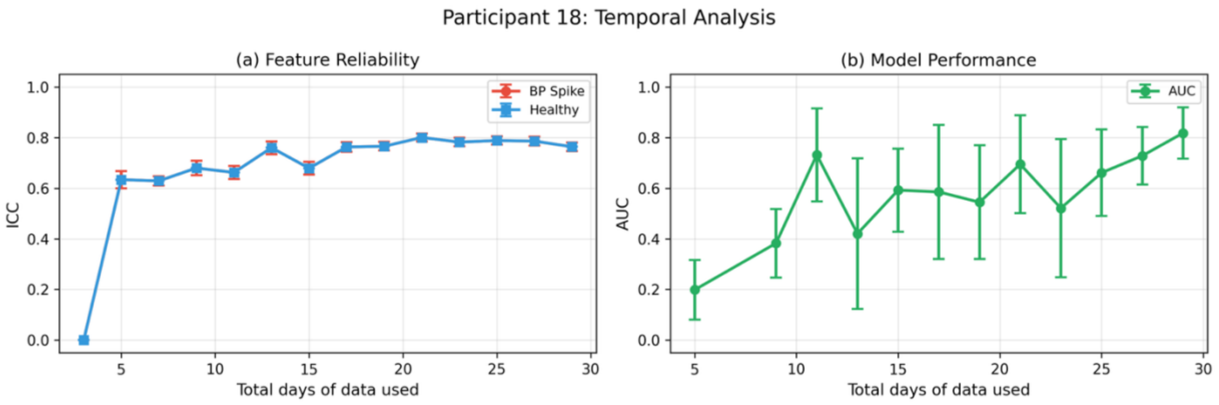
Participant 18: Temporal Analysis
(a) Feature Reliability
ICC
BP Spike
Healthy
Total days of data used
(b) Model Performance
AUC
AUC
Total days of data used

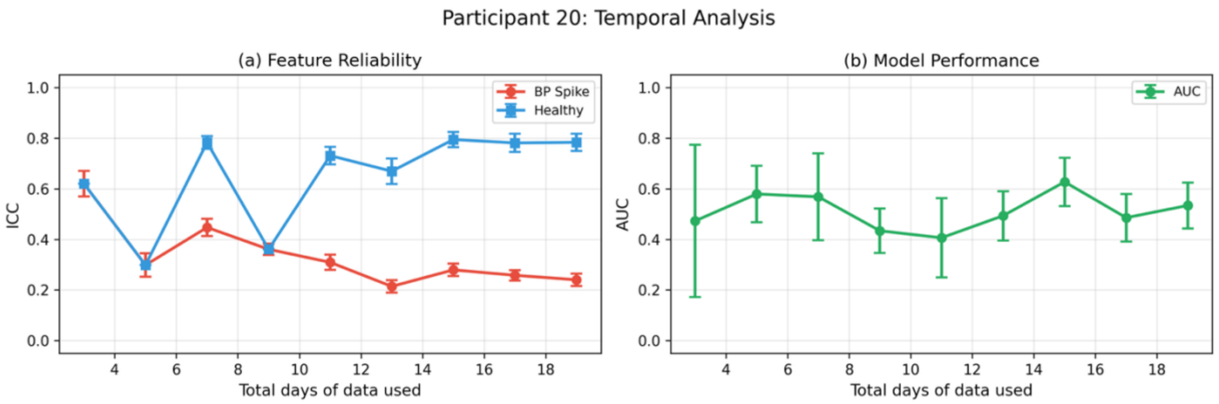
Participant 20: Temporal Analysis
(a) Feature Reliability
ICC
BP Spike
Healthy
Total days of data used
(b) Model Performance
AUC
AUC
Total days of data used

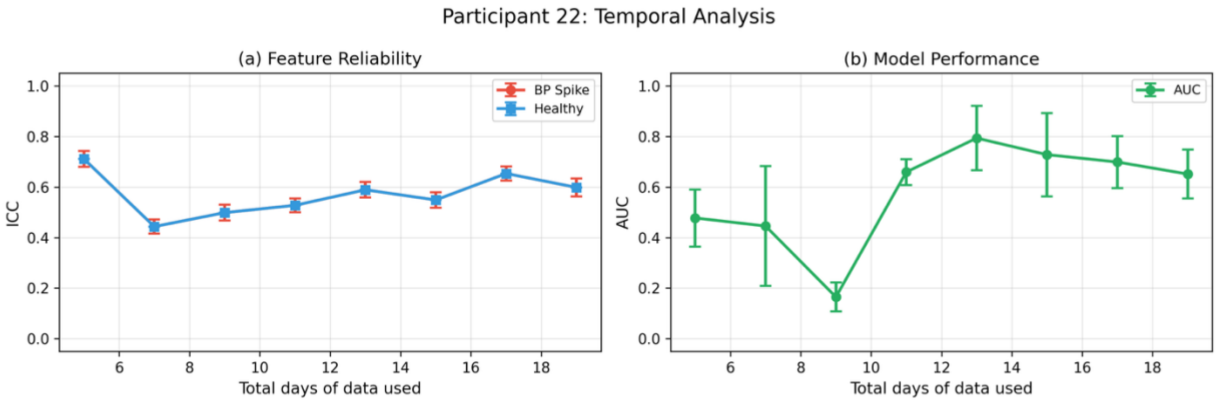
Participant 22: Temporal Analysis
(a) Feature Reliability
BP Spike
Healthy
ICC
Total days of data used
(b) Model Performance
AUC
Total days of data used

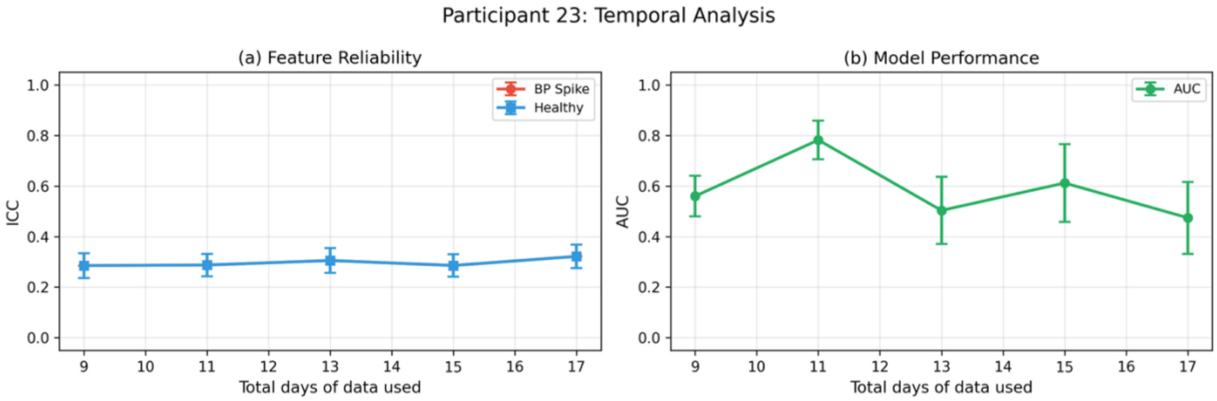
Participant 23: Temporal Analysis
(a) Feature Reliability
BP Spike
Healthy
ICC
Total days of data used
(b) Model Performance
AUC
Total days of data used

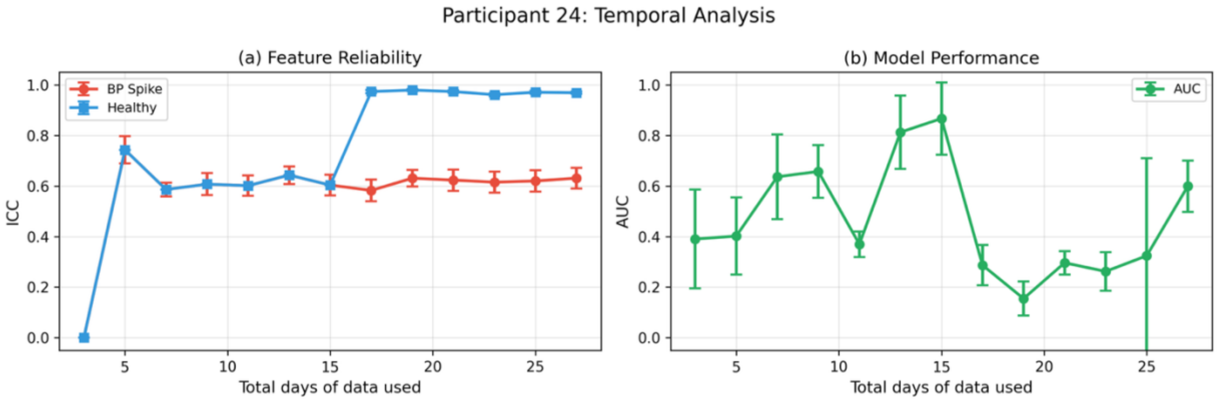
Participant 24: Temporal Analysis
(a) Feature Reliability
BP Spike
Healthy
ICC
Total days of data used
(b) Model Performance
AUC
Total days of data used

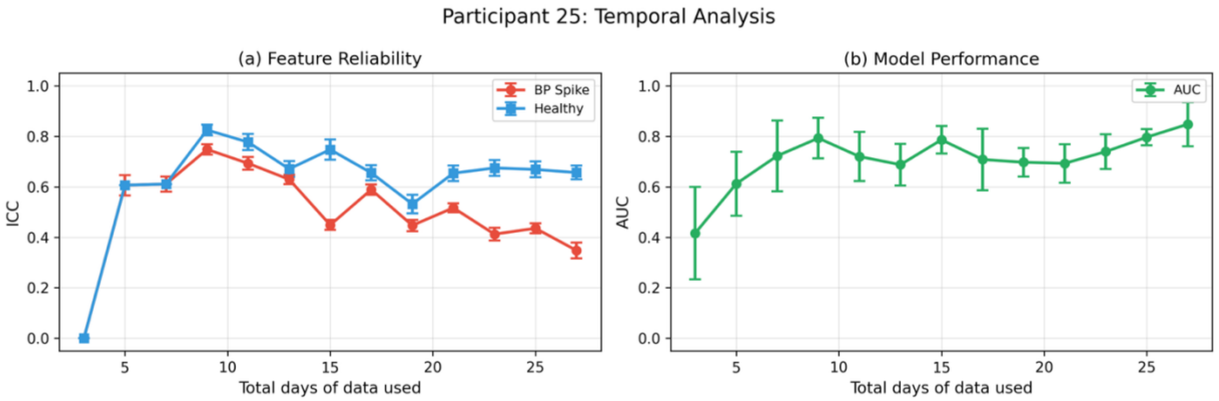
Participant 25: Temporal Analysis
(a) Feature Reliability
BP Spike
Healthy
ICC
1.0
0.8
0.6
0.4
0.2
0.0
5
10
15
20
25
Total days of data used
(b) Model Performance
AUC
AUC
1.0
0.8
0.6
0.4
0.2
0.0
5
10
15
20
25
Total days of data used

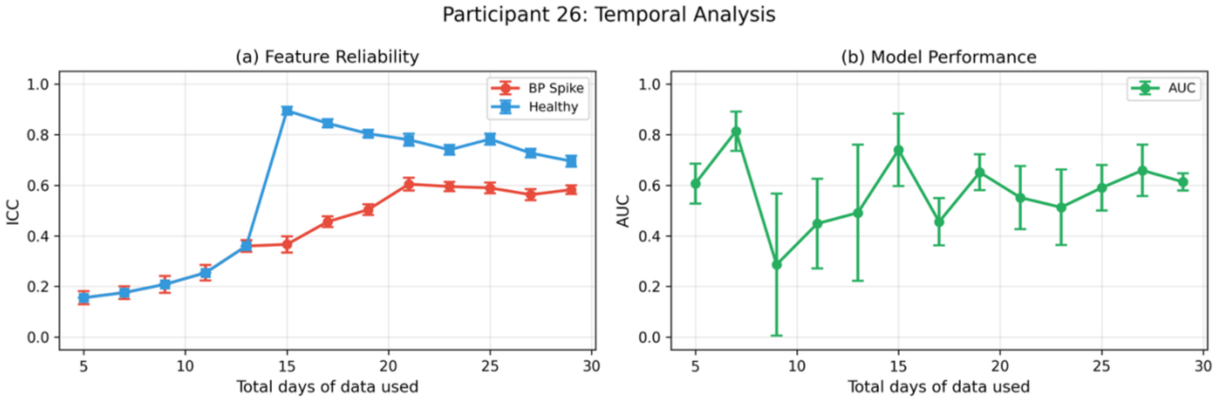
Participant 26: Temporal Analysis
(a) Feature Reliability
BP Spike
Healthy
ICC
1.0
0.8
0.6
0.4
0.2
0.0
5
10
15
20
25
30
Total days of data used
(b) Model Performance
AUC
AUC
1.0
0.8
0.6
0.4
0.2
0.0
5
10
15
20
25
30
Total days of data used

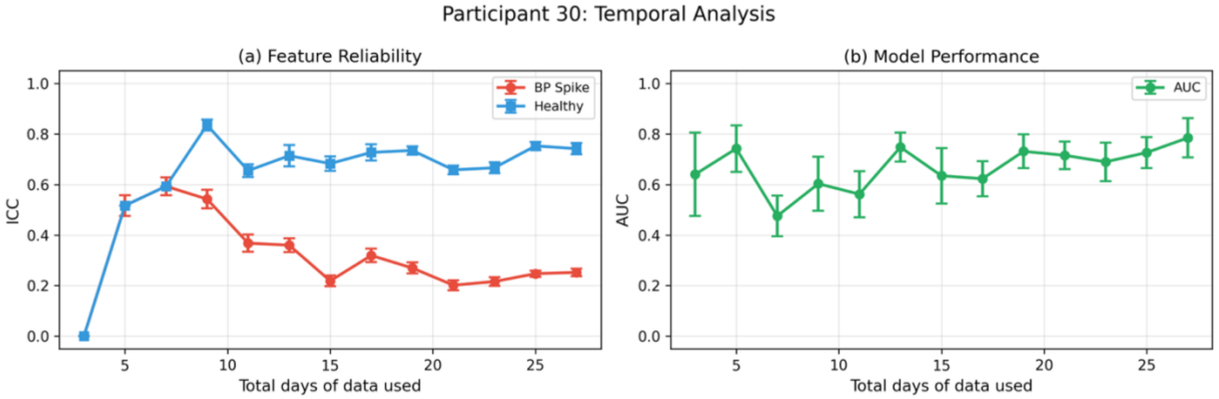
Participant 30: Temporal Analysis
(a) Feature Reliability
BP Spike
Healthy
ICC
1.0
0.8
0.6
0.4
0.2
0.0
5
10
15
20
25
Total days of data used
(b) Model Performance
AUC
AUC
1.0
0.8
0.6
0.4
0.2
0.0
5
10
15
20
25
Total days of data used

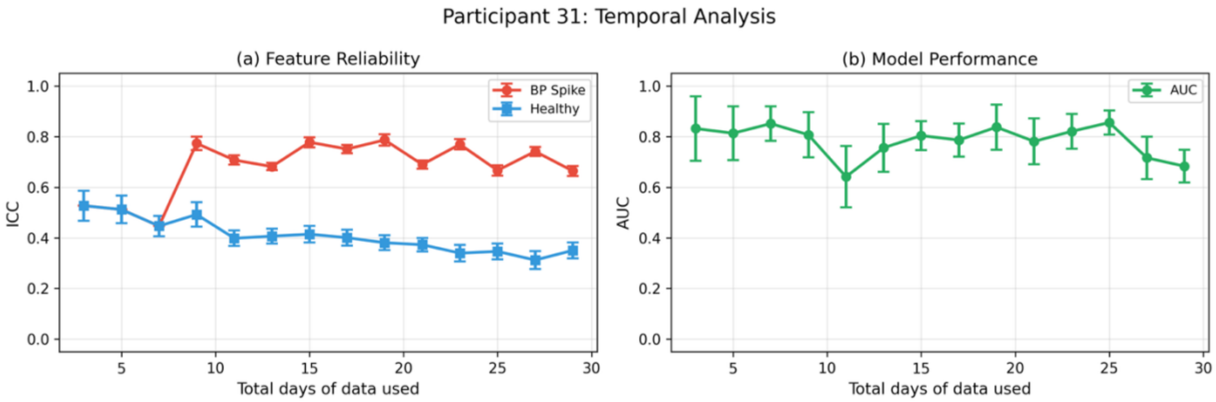
Participant 31: Temporal Analysis
(a) Feature Reliability
BP Spike
Healthy
ICC
Total days of data used
(b) Model Performance
AUC
AUC
Total days of data used

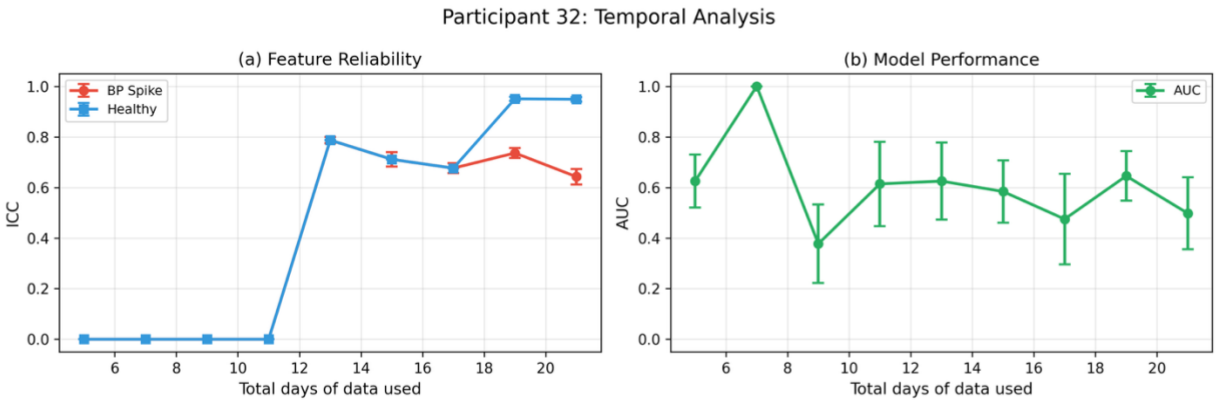
Participant 32: Temporal Analysis
(a) Feature Reliability
BP Spike
Healthy
ICC
Total days of data used
(b) Model Performance
AUC
AUC
Total days of data used

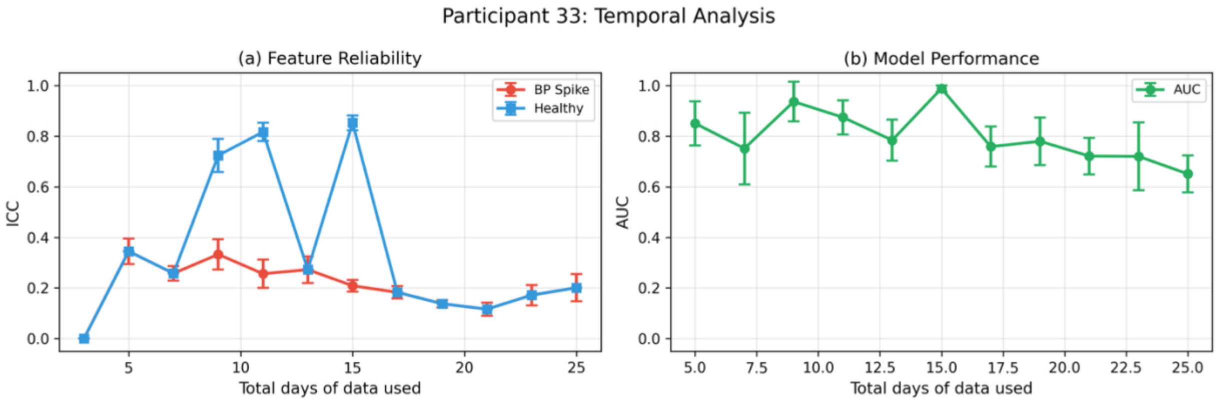
Participant 33: Temporal Analysis
(a) Feature Reliability
BP Spike
Healthy
ICC
Total days of data used
(b) Model Performance
AUC
AUC
Total days of data used

Participant 34: Temporal Analysis

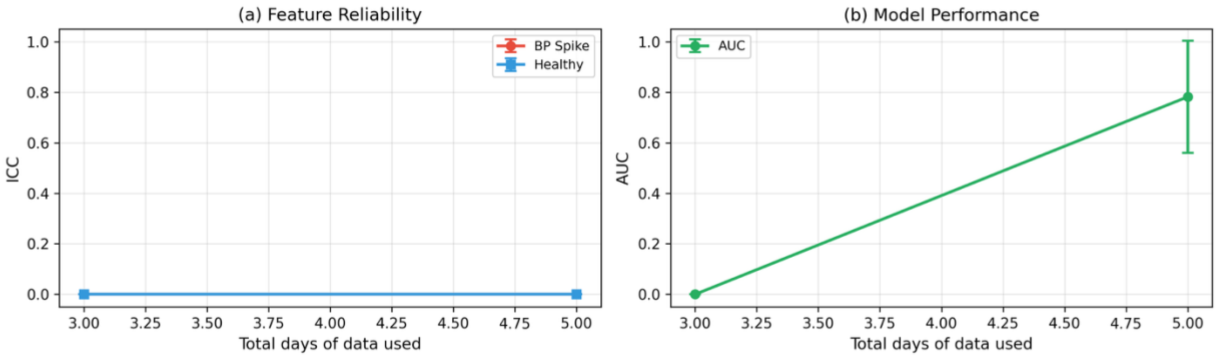

Participant 35: Temporal Analysis

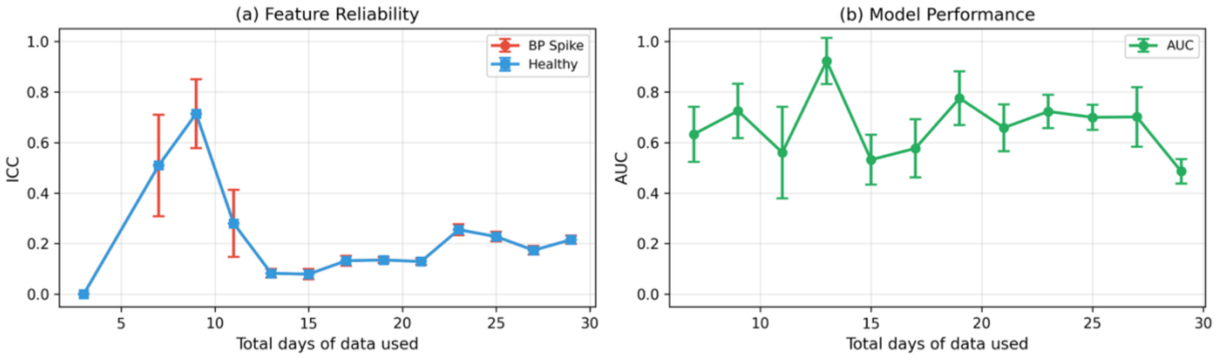

Participant 36: Temporal Analysis

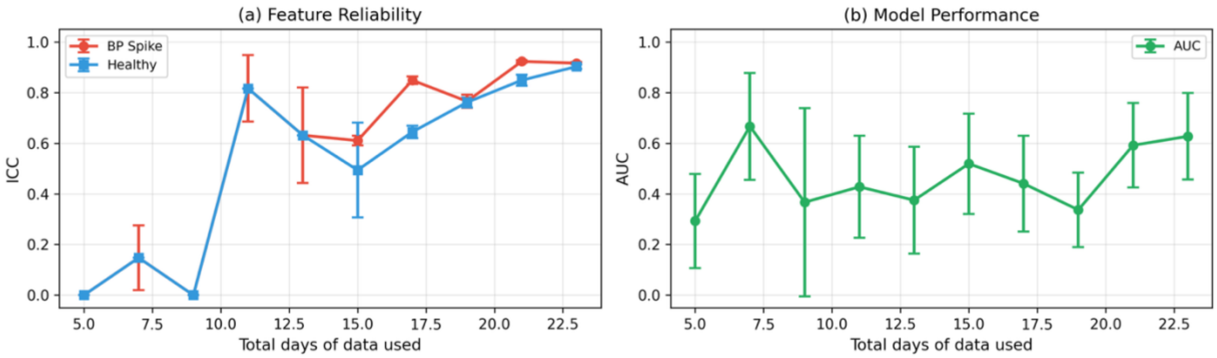

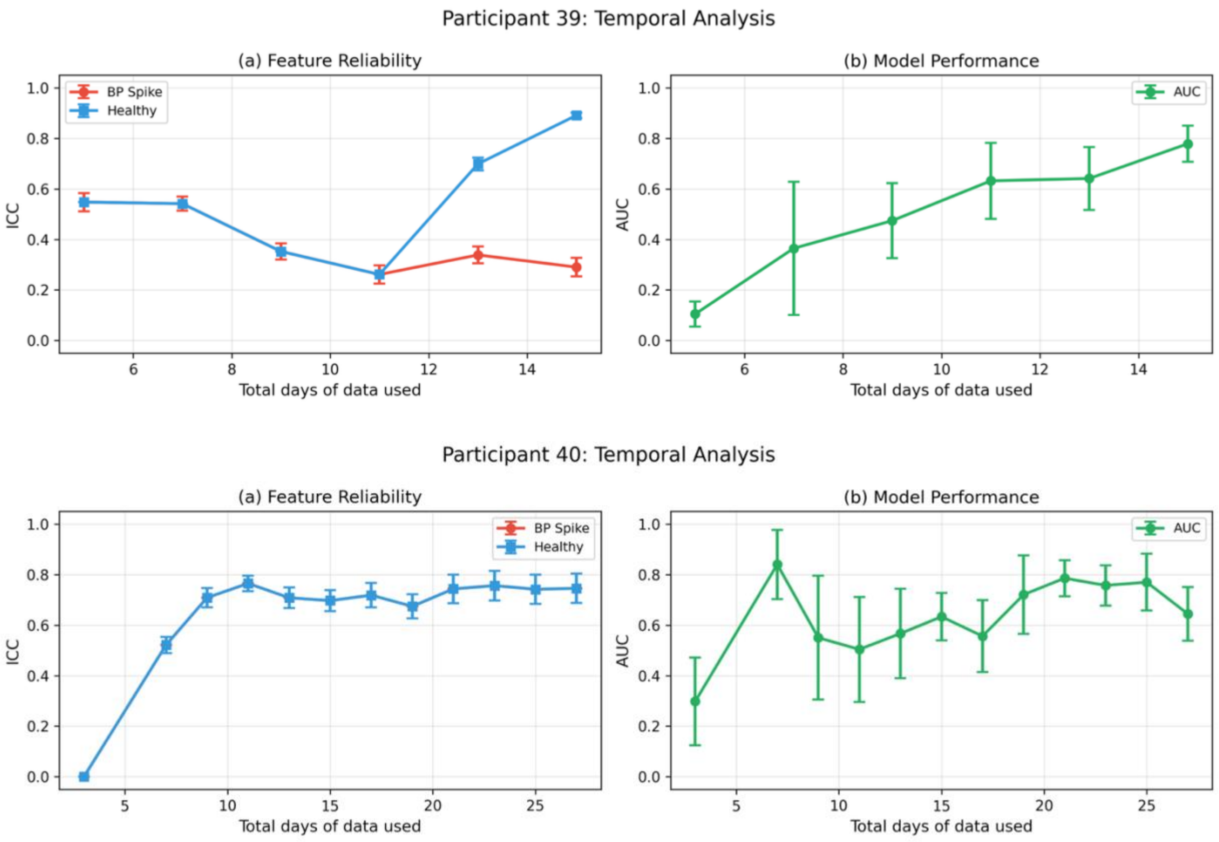

Figure 4. ICC Analysis of Feature Reliability and Model Performance of Personalized Models (IDs increasing row-wise). Participants 17, 32, 39 were excluded owing to insufficient labels.

Personalized temporal analyses (Figure 4) revealed substantial inter-individual heterogeneity in data sufficiency and model stability across participants. Several participants lacked ICC estimates for early durations (3-7 days) due to insufficient elevated BP events to satisfy the minimum requirements for reliable estimation. For example, Participant 22 shows limited early ICC data points, indicating lower event frequency in initial monitoring periods, while Participant 25 demonstrates stable ICC values from early timepoints with higher event rates. Performance trajectories varied markedly: some participants like Participant 26 showed declining AUC with extended training periods, potentially due to overfitting or temporal drift, while others like Participant 30 maintained consistent performance across all durations.

Across the cohort, 7-11 days of monitoring appeared sufficient for most participants to achieve stable model performance, however, those with lower elevated BP prevalence or irregular device usage patterns required 15-23 days for reliable personalized model development. The heterogeneous patterns highlight the importance of adaptive monitoring strategies tailored to individual elevated BP frequency and usage adherence. Participants with higher event rates demonstrated rapid performance stabilization, supporting personalized onboarding protocols that could optimize monitoring duration based on early performance indicators and event capture rates in real-world deployment scenarios.

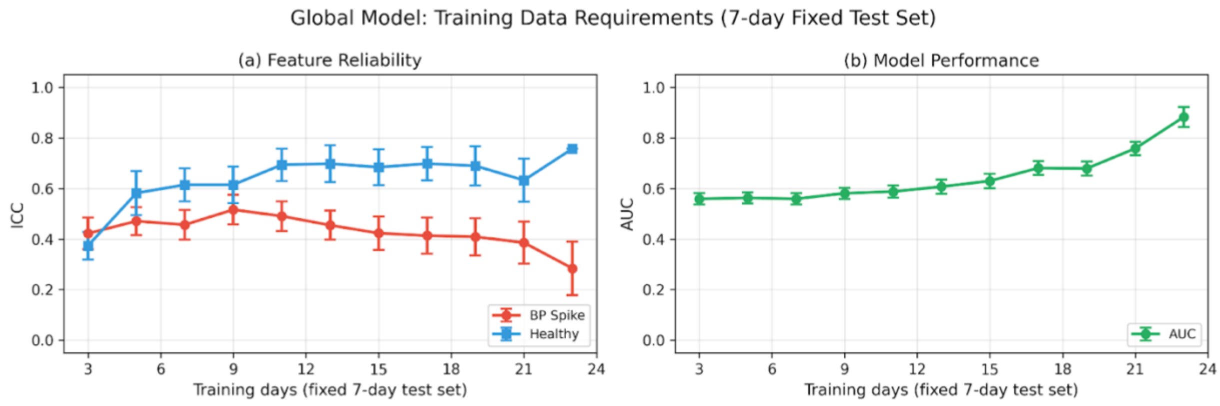

Figure 5. ICC Analysis of (a) Feature Reliability and (b) Model Performance of Global Model using 7-day Fixed Test Set

While the prior analysis showed shorter study periods having negligible impact on model performance, there still was a need to address the distinct question of what were the minimum training data requirements for reliable predictions with a proper, fixed test dataset. We thus conducted a complementary temporal analysis using a fixed 7-day test set comprising the final week of each participant's monitoring period. Unlike the original analysis which varied total monitoring duration with proportional train-test splits, this approach isolates the effect of training set size on model performance by holding the test set constant. Training days ranged from 3 to 29 (where maximum = total days - 7), using only odd-numbered days to reduce computational burden while maintaining adequate resolution.

For the global model with fixed test evaluation (Figure 5a), feature reliability metrics demonstrated divergent trajectories between elevated BP and healthy states. Elevated BP ICC values started at approximately 0.45 ± 0.08 at 3 days and showed moderate stability around 0.47 ± 0.05 through 13 days, before declining progressively to 0.28 ± 0.14 at 25 days. This degradation at longer durations likely reflects the decreasing sample size of participants with sufficient monitoring periods, as participants with shorter engagement periods are systematically excluded from later training windows, reducing both statistical power and population representativeness. Conversely, healthy-state ICC improved substantially from 0.37 ± 0.09 with minimal training to 0.76 ± 0.07 at 25 days, demonstrating that baseline physiological patterns require extended observation periods for stable characterization. The global model's predictive performance showed substantial improvement with increased training data (Figure 5b), rising from AUC = 0.56 ± 0.03 at 3 days to 0.89 ± 0.04 at 23 days, indicating that adequate training duration is critical when evaluated against a fixed test period.

Participant 10: Training Data Requirements (7-day test)

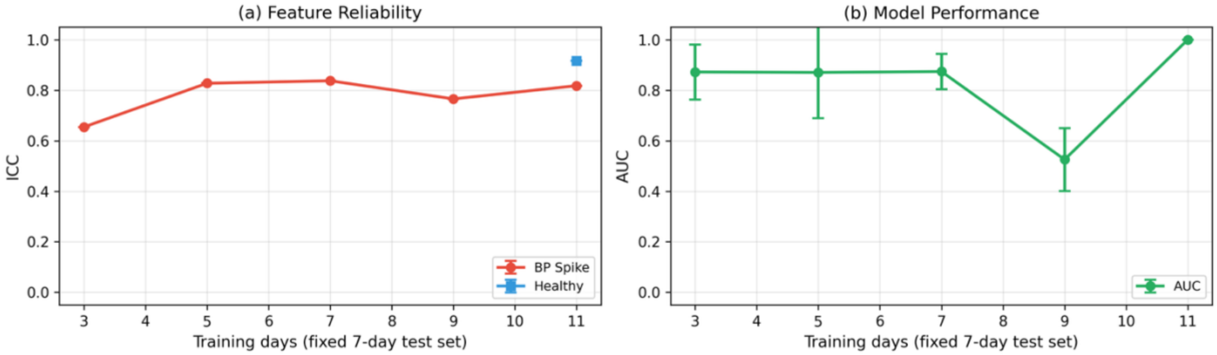

Participant 15: Training Data Requirements (7-day test)

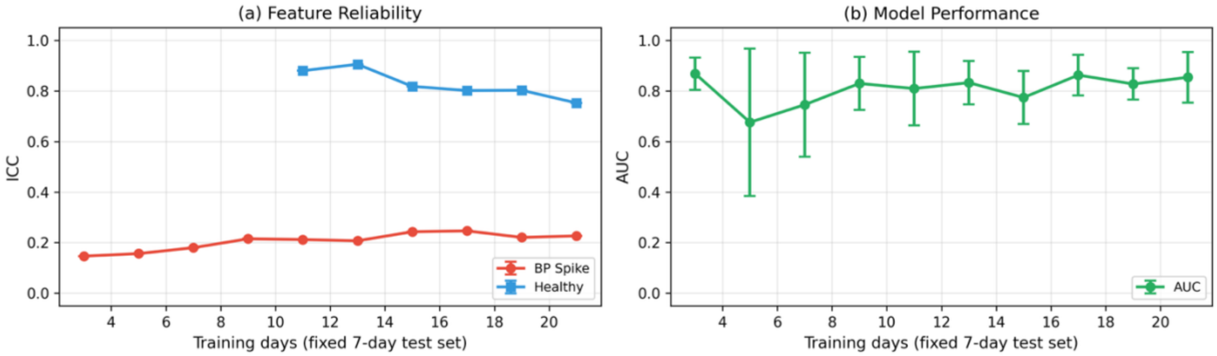

Participant 16: Training Data Requirements (7-day test)

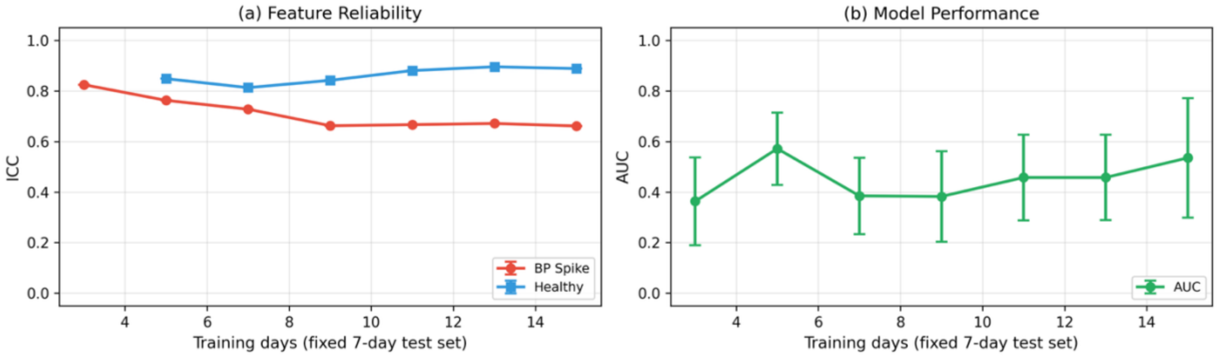

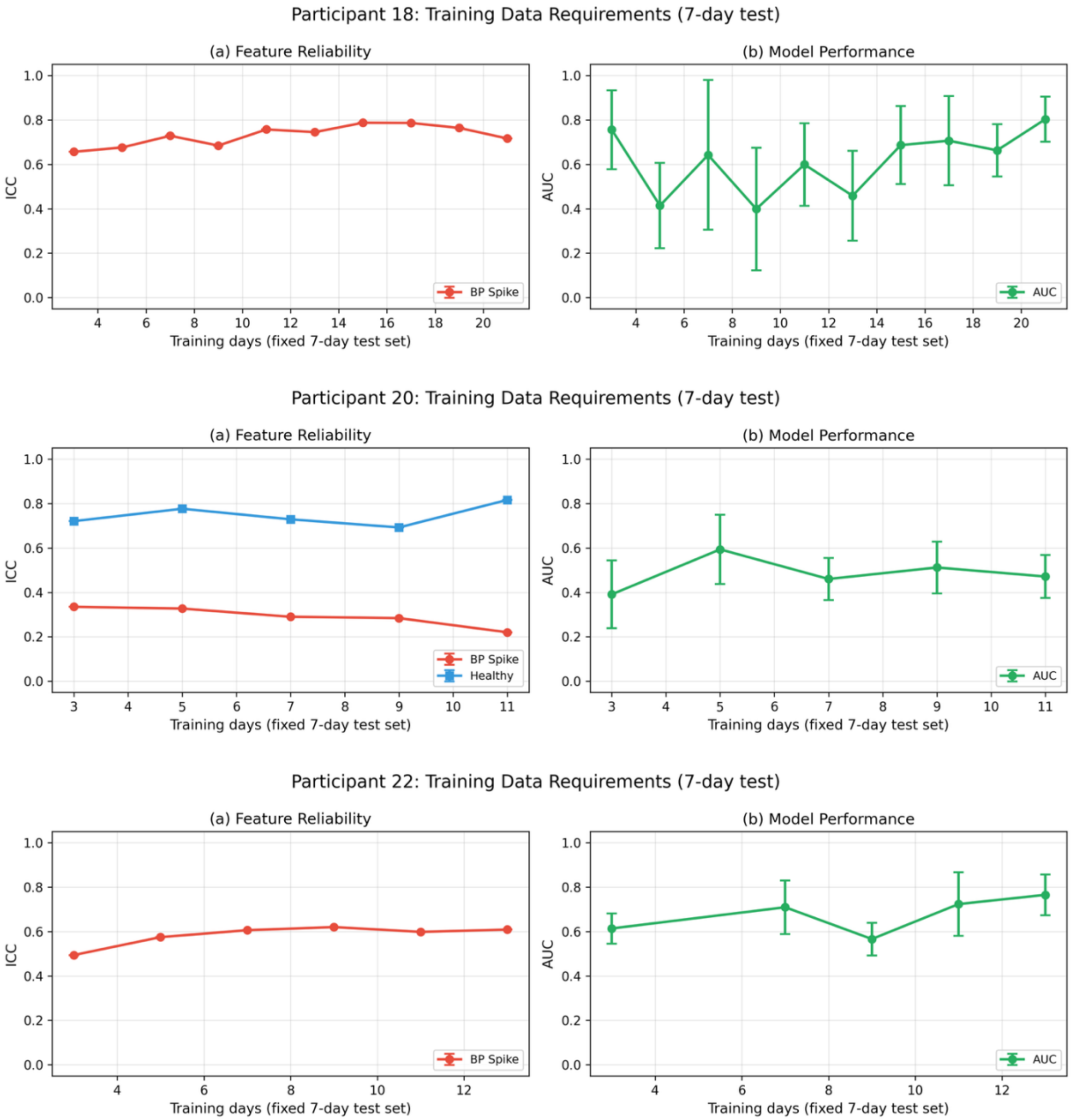
Participant 18: Training Data Requirements (7-day test)
(a) Feature Reliability
ICC
BP Spike
Training days (fixed 7-day test set)
(b) Model Performance
AUC
AUC
Training days (fixed 7-day test set)
Participant 20: Training Data Requirements (7-day test)
(a) Feature Reliability
ICC
BP Spike
Healthy
Training days (fixed 7-day test set)
(b) Model Performance
AUC
AUC
Training days (fixed 7-day test set)
Participant 22: Training Data Requirements (7-day test)
(a) Feature Reliability
ICC
BP Spike
Training days (fixed 7-day test set)
(b) Model Performance
AUC
AUC
Training days (fixed 7-day test set)

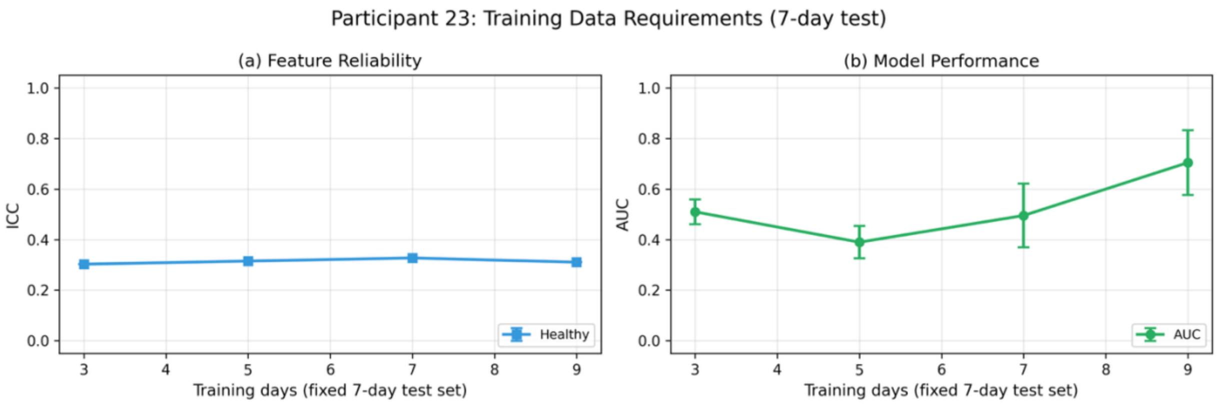
Participant 23: Training Data Requirements (7-day test)
(a) Feature Reliability
ICC
Healthy
Training days (fixed 7-day test set)
(b) Model Performance
AUC
AUC
Training days (fixed 7-day test set)

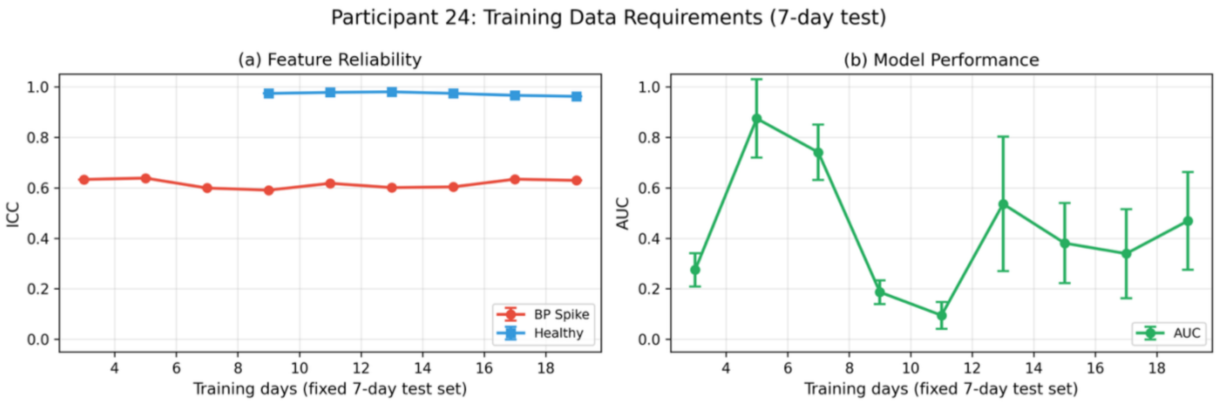
Participant 24: Training Data Requirements (7-day test)
(a) Feature Reliability
ICC
BP Spike
Healthy
Training days (fixed 7-day test set)
(b) Model Performance
AUC
AUC
Training days (fixed 7-day test set)

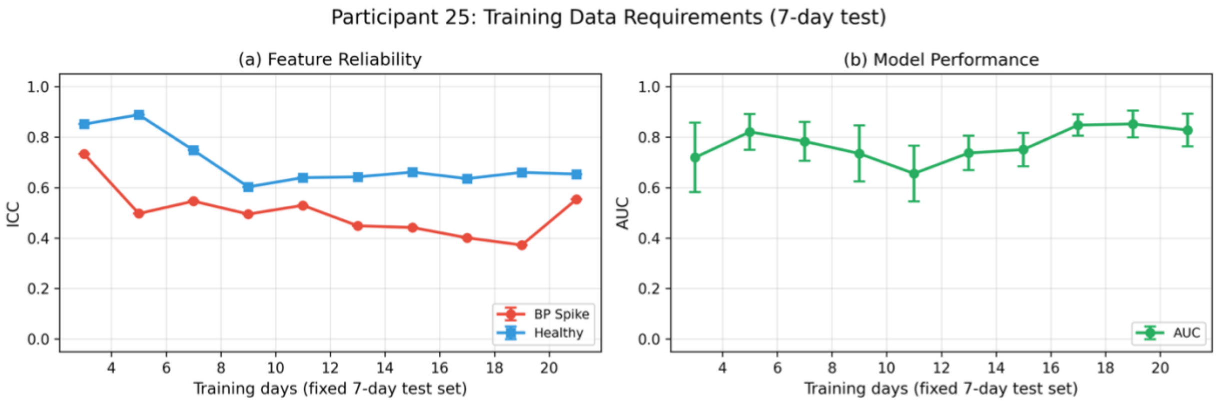
Participant 25: Training Data Requirements (7-day test)
(a) Feature Reliability
ICC
BP Spike
Healthy
Training days (fixed 7-day test set)
(b) Model Performance
AUC
AUC
Training days (fixed 7-day test set)

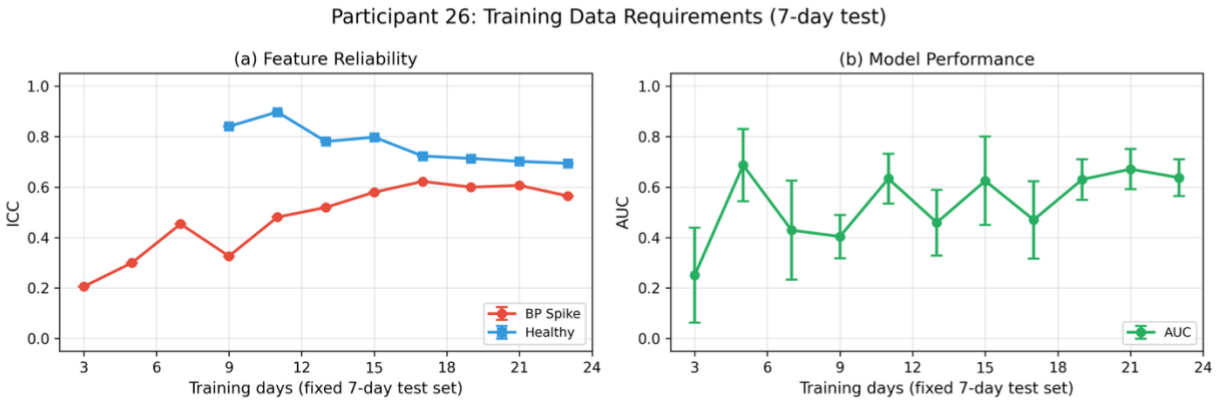
Participant 26: Training Data Requirements (7-day test)
(a) Feature Reliability
ICC
BP Spike
Healthy
Training days (fixed 7-day test set)
(b) Model Performance
AUC
AUC
Training days (fixed 7-day test set)

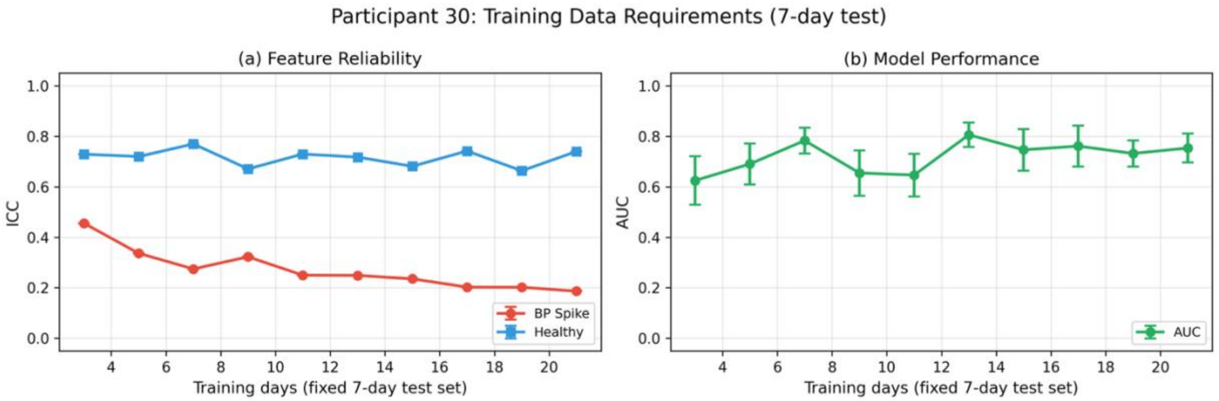
Participant 30: Training Data Requirements (7-day test)
(a) Feature Reliability
ICC
BP Spike
Healthy
Training days (fixed 7-day test set)
(b) Model Performance
AUC
AUC
Training days (fixed 7-day test set)

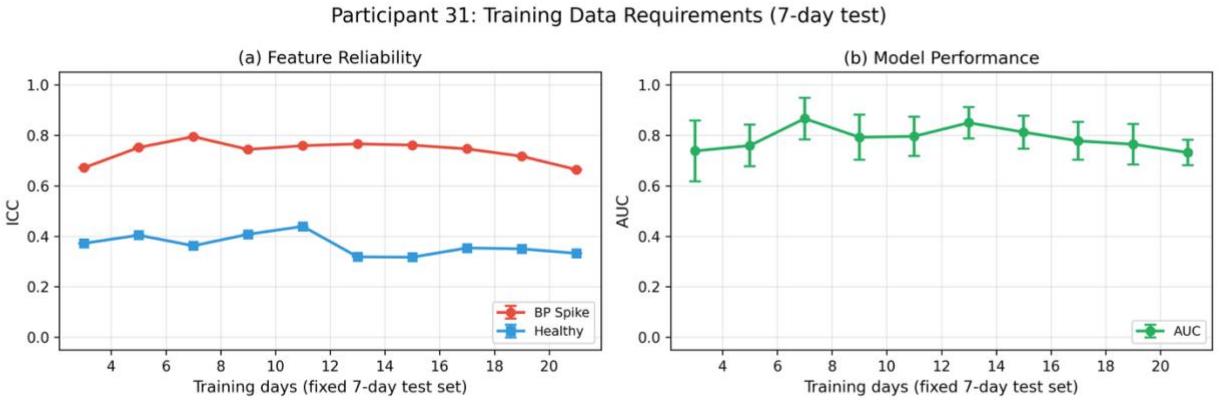
Participant 31: Training Data Requirements (7-day test)
(a) Feature Reliability
ICC
BP Spike
Healthy
Training days (fixed 7-day test set)
(b) Model Performance
AUC
AUC
Training days (fixed 7-day test set)

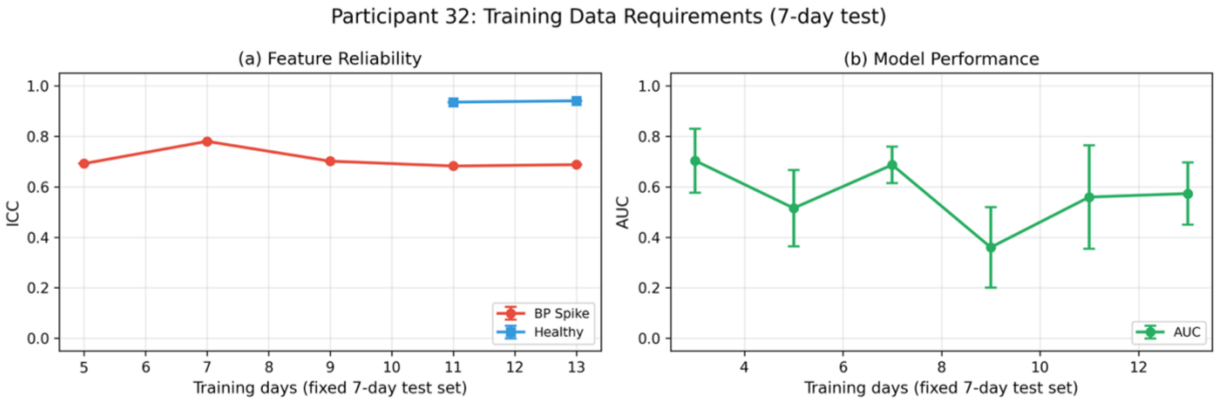
Participant 32: Training Data Requirements (7-day test)
(a) Feature Reliability
ICC
Training days (fixed 7-day test set)
BP Spike
Healthy
(b) Model Performance
AUC
Training days (fixed 7-day test set)
AUC

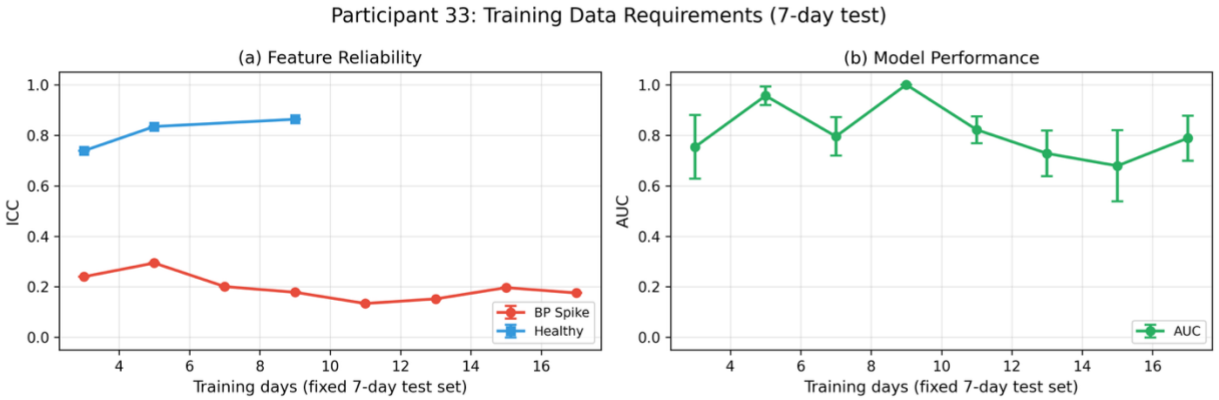
Participant 33: Training Data Requirements (7-day test)
(a) Feature Reliability
ICC
Training days (fixed 7-day test set)
BP Spike
Healthy
(b) Model Performance
AUC
Training days (fixed 7-day test set)
AUC

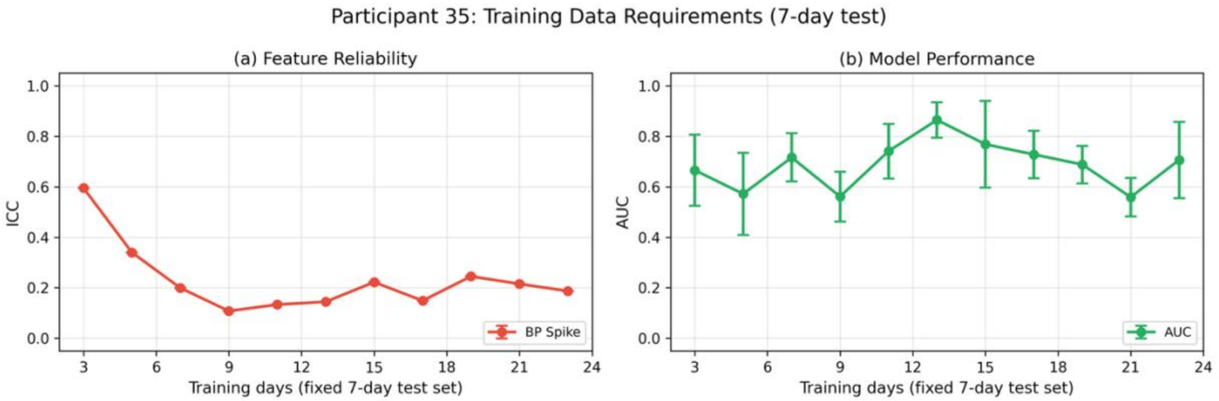
Participant 35: Training Data Requirements (7-day test)
(a) Feature Reliability
ICC
Training days (fixed 7-day test set)
BP Spike
(b) Model Performance
AUC
Training days (fixed 7-day test set)
AUC

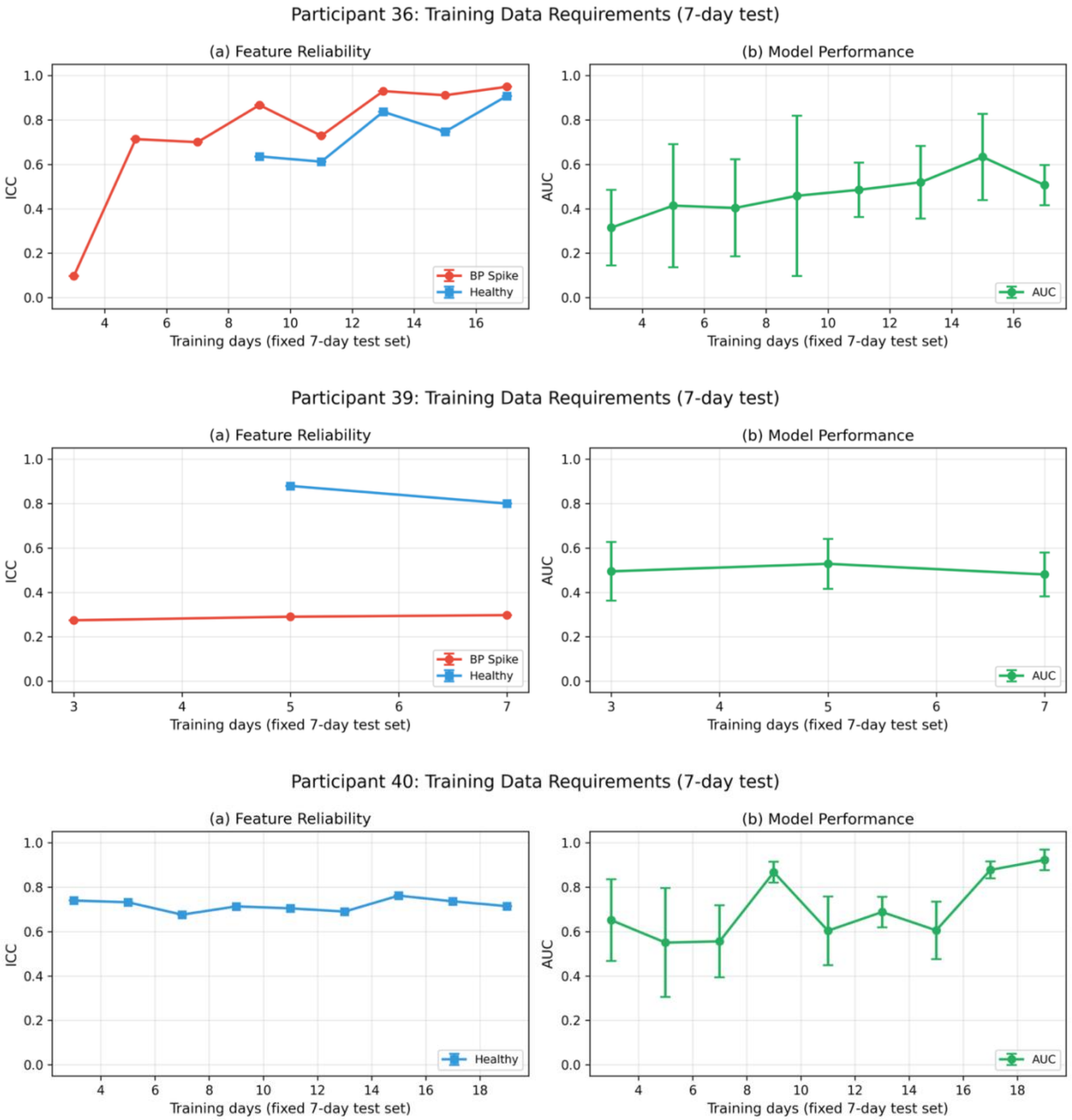

Figure 6. ICC Analysis of Feature Reliability and Model Performance of Personalized Models (IDs increasing row-wise) using 7-day Fixed Test Set. Participant 34 was excluded owing to insufficient labels.

Personalized analyses with fixed 7-day test sets (Figure 6) revealed marked heterogeneity in minimum training requirements and temporal stability across the cohort, with two distinct phenotypes emerging. High-performing rapid learners achieved near-optimal predictive accuracy with minimal training data. Participant 10 demonstrated exceptional early learning, reaching AUROC of approximately ≈0.90 with just 3 days of training and maintaining ≈0.88–0.92 across subsequent training durations. Participant 16 showed a similar trajectory, starting around 0.75 at 3 days and rapidly improving to ≈0.85–0.90 by days 7–9, maintaining stable performance thereafter. Participant 22 exhibited strong performance (AUROC of

approximately ≈0.80–0.85) with moderate fluctuation across the training horizon. These rapid learners suggest that individuals with regular elevated BP patterns and relatively high event frequency can be effectively modeled with as little as 5–7 days of personalized training.

By contrast, a substantial subset of participants appeared data-limited or intrinsically harder to model under the fixed-window paradigm. Participants such as 18, 20, and 24 exhibited AUC curves that remained close to or below chance (≈0.40–0.60) despite increasing training duration, often with wide confidence intervals and no clear monotonic improvement. Participant 26 showed declining performance from approximately 0.70 at early training days to near-chance levels at longer durations, potentially indicating temporal drift or overfitting. This pattern is consistent with insufficient elevated BP events, irregular monitoring, or strong temporal non-stationarities that violate the assumptions of a fixed 7-day test window. The higher prevalence of these "data-insufficient" profiles compared with the proportional-split analysis underscores the more stringent data requirements of fixed-window prediction and highlights potential barriers to real-world deployment when monitoring is brief or sporadic.

Feature-reliability metrics further revealed divergent patterns between elevated BP and healthy states across individuals. Participant 30 showed pronounced asymmetry, with healthy-state ICC values rising to 0.7–0.8 range at longer training durations, while elevated BP ICC remained relatively lower and more variable (0.3–0.5), suggesting highly consistent baseline physiology but more variable elevated BP characteristics. Conversely, Participant 23 exhibited relatively stable but modest ICC values for both conditions, with elevated BP ICC showing gradual improvement to 0.6–0.7 while healthy-state ICC remained around 0.4–0.5. Participant 33 displayed strong healthy-state ICC (0.7–0.8) with more variable elevated BP ICC patterns. These contrasting reliability profiles imply that, for some individuals, improving elevated BP detection may require modeling baseline variability, whereas for others it may require richer characterization of elevated BP episodes themselves.

Overall, these findings refine our original temporal analysis, which suggested relatively uniform requirements of ~7–10 days of data across participants. Under a fixed 7-day test regime, many participants still achieve reliable predictions with only 5–9 training days, but a sizable minority never attain stable, above-chance performance even after their full monitoring period. This heterogeneity argues for adaptive onboarding protocols that explicitly account for individual elevated BP frequency, temporal stability, and adherence patterns, allowing rapid deployment for high-frequency responders, while signaling the need for extended observation windows (e.g., 14–21 days) or alternative modeling strategies in participants with sparse events or pronounced temporal drift.

Attention-weight profiles from the BiLSTM models (Figure 7) concentrate on short windows (5–10 min) while retaining non-trivial weight on longer windows (30–60 min), indicating that the sequence models integrate rapid physiological changes with slower contextual dynamics. Wide 95% CIs across participants highlight substantial inter-individual differences in the temporal patterns the model relies upon, reinforcing the need for personalization.

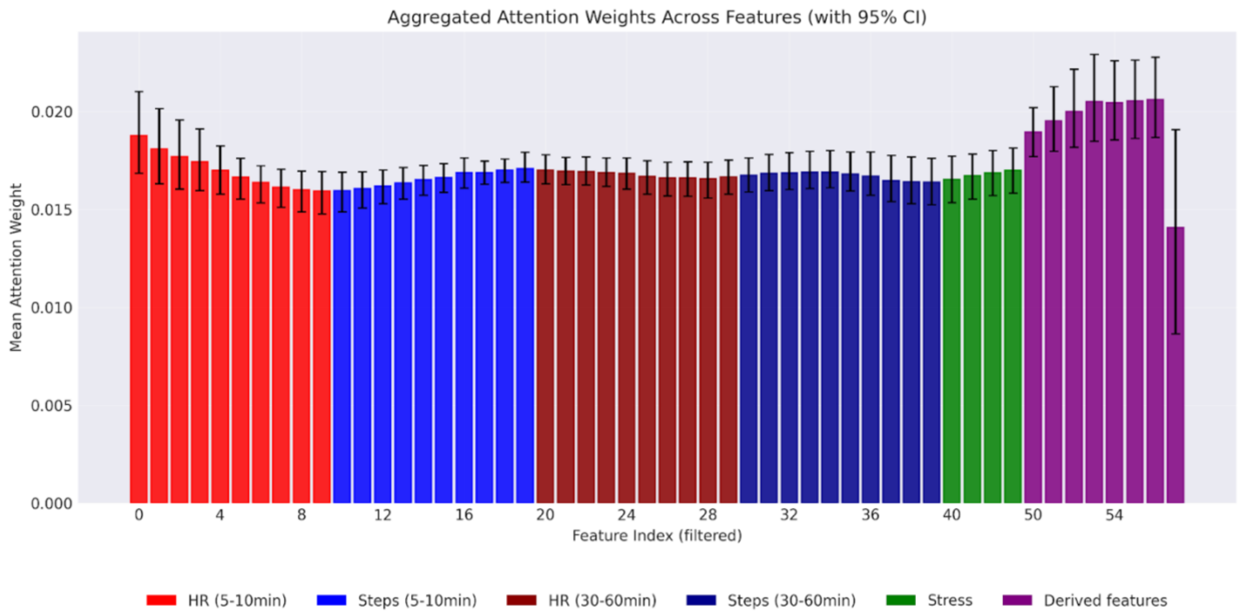

Figure 7. Aggregated attention weights across feature categories.

Table 1. Participant-specific model configurations and performance for BP prediction

PID	Best Pipeline	α	XGB Depth	XGB LR	XGB Estimators	ADASYN Sampling	LSTM Units (L1/L2)	LSTM LR	Dropout	Attention
**10**	LSTM	0.0	3	0.01	150	0.70	128/128	0.0005	0.2	self-attention
**15**	LSTM	0.0	3	0.01	150	0.75	64/128	0.001	0.4	multi-head
**16**	Ensemble	0.2	5	0.01	150	—	256/112	0.001	0.3	custom
**18**	LSTM	0.0	3	0.05	100	0.60	96/48	0.0005	0.3	self-attention
**20**	LSTM	0.0	3	0.10	100	0.70	128/48	0.001	0.4	custom
**22**	Ensemble	0.2	3	0.01	100	0.75	192/48	0.001	0.2	self-attention
**23**	Ensemble	0.4	3	0.05	100	0.75	256/32	0.001	0.4	self-attention
**24**	Ensemble	0.1	5	0.05	200	0.70	96/96	0.0005	0.2	multi-head
**25**	Ensemble	0.3	3	0.05	200	—	96/48	0.001	0.4	multi-head
**26**	LSTM	0.0	5	0.01	100	0.60	256/128	0.0005	0.2	custom
**30**	Ensemble	0.6	3	0.05	100	0.65	192/128	0.0005	0.4	custom
**31**	LSTM	0.0	3	0.01	200	0.70	96/96	0.001	0.2	self-attention
**32**	Ensemble	0.1	3	0.01	100	—	224/32	0.001	0.3	multi-head
**33**	LSTM	0.0	3	0.01	200	0.60	192/128	0.001	0.2	multi-head
**34**	Ensemble	0.5	3	0.01	100	—	64/96	0.0001	0.3	custom
**35**	Ensemble	0.3	3	0.01	100	0.60	128/64	0.0001	0.4	custom
**36**	LSTM	0.1	5	0.10	200	—	224/112	0.0001	0.2	custom
**39**	Ensemble	0.5	5	0.01	100	0.75	224/112	0.0005	0.2	multi-head
**40**	LSTM	0.0	3	0.01	100	0.60	192/64	0.001	0.2	self-attention

Table 2. Participant-specific model configurations and performance for high-stress prediction

PID	Best Pipeline	α	XGB Depth	XGB LR	XGB Estimators	ADASYN Sampling	LSTM Units (L1/L2)	LSTM LR	Dropout	Attention
**10**	LSTM	0.0	3	0.01	150	0.70	64 / 64	0.001	0.3	custom
**15**	Ensemble	0.1	3	0.01	200	—	128 / 48	0.0001	0.3	custom
**16**	Ensemble	0.2	3	0.05	100	0.60	256 / 32	0.0005	0.3	multi-head
**18**	LSTM	0.0	3	0.10	100	—	224 / 80	0.001	0.2	self-attention
**20**	LSTM	0.0	3	0.10	150	—	96 / 128	0.001	0.4	multi-head
**22**	LSTM	0.0	3	0.01	100	—	160 / 128	0.0005	0.2	multi-head
**23**	LSTM	0.0	3	0.01	200	0.60	224 / 112	0.0005	0.2	custom
**24**	Ensemble	0.3	3	0.01	200	0.75	256 / 96	0.001	0.2	custom
**25**	Ensemble	0.5	5	0.01	100	0.70	128 / 96	0.0005	0.2	multi-head
**26**	LSTM	0.0	5	0.01	200	0.65	160 / 96	0.0001	0.3	custom
**30**	LSTM	0.0	7	0.10	200	0.70	64 / 128	0.0001	0.3	custom
**31**	Ensemble	0.1	7	0.05	150	0.60	256 / 80	0.001	0.4	self-attention
**34**	Ensemble	0.1	3	0.01	100	—	256 / 80	0.0001	0.2	self-attention
**35**	LSTM	0.0	3	0.01	100	0.60	160 / 112	0.0005	0.4	custom
**36**	Ensemble	0.2	3	0.01	200	0.70	256 / 112	0.001	0.3	custom
**39**	LSTM	0.0	3	0.05	100	—	256 / 128	0.001	0.4	multi-head

Table 3. AUROC (mean ± standard deviation) for participant-specific models under feature ablation. Each column shows performance after removing a feature family, enabling comparison of their relative contributions to predictive performance.

PID	Cumulative	HR features	Ratios	Step features	Stress features	Time features
**hp10**	0.818 ± 0.091	0.858 ± 0.061	0.846 ± 0.077	0.851 ± 0.065	0.743 ± 0.070	0.825 ± 0.073
**hp15**	0.754 ± 0.047	0.737 ± 0.049	0.732 ± 0.049	0.717 ± 0.056	0.613 ± 0.059	0.761 ± 0.051
**hp16**	0.639 ± 0.081	0.553 ± 0.087	0.721 ± 0.082	0.765 ± 0.086	0.629 ± 0.085	0.707 ± 0.079
**hp18**	0.798 ± 0.142	0.886 ± 0.099	0.859 ± 0.124	0.885 ± 0.070	0.759 ± 0.181	0.813 ± 0.091
**hp20**	0.951 ± 0.050	0.917 ± 0.070	0.902 ± 0.082	0.917 ± 0.054	0.950 ± 0.063	1.000 ± 0.000
**hp22**	0.869 ± 0.065	0.842 ± 0.092	0.733 ± 0.129	0.884 ± 0.059	0.757 ± 0.131	0.895 ± 0.083
**hp23**	0.909 ± 0.089	0.760 ± 0.155	0.824 ± 0.109	0.809 ± 0.094	0.883 ± 0.055	0.839 ± 0.084
**hp24**	0.694 ± 0.111	0.692 ± 0.120	0.692 ± 0.110	0.539 ± 0.204	0.751 ± 0.099	0.753 ± 0.098
**hp25**	0.817 ± 0.074	0.808 ± 0.080	0.783 ± 0.084	0.815 ± 0.071	0.675 ± 0.093	0.801 ± 0.084
**hp26**	0.639 ± 0.090	0.650 ± 0.089	0.597 ± 0.105	0.650 ± 0.093	0.588 ± 0.104	0.670 ± 0.091
**hp30**	0.766 ± 0.071	0.745 ± 0.064	0.756 ± 0.067	0.718 ± 0.082	0.758 ± 0.068	0.759 ± 0.068
**hp31**	0.711 ± 0.118	0.711 ± 0.106	0.733 ± 0.117	0.674 ± 0.130	0.762 ± 0.108	0.721 ± 0.112
**hp32**	0.655 ± 0.111	0.686 ± 0.104	0.677 ± 0.110	0.703 ± 0.104	0.625 ± 0.115	0.601 ± 0.112
**hp33**	0.826 ± 0.108	0.844 ± 0.100	0.760 ± 0.135	0.510 ± 0.209	0.815 ± 0.113	0.883 ± 0.097
**hp34**	0.793 ± 0.127	0.895 ± 0.094	1.000 ± 0.000	0.844 ± 0.121	0.945 ± 0.058	1.000 ± 0.000
**hp35**	0.775 ± 0.096	0.713 ± 0.126	0.807 ± 0.104	0.749 ± 0.101	0.846 ± 0.111	0.811 ± 0.106
**hp36**	0.950 ± 0.068	1.000 ± 0.000	1.000 ± 0.000	0.876 ± 0.117	1.000 ± 0.000	0.950 ± 0.074
**hp39**	1.000 ± 0.000	1.000 ± 0.000	1.000 ± 0.000	1.000 ± 0.000	1.000 ± 0.000	1.000 ± 0.000
**hp40**	0.950 ± 0.073	0.954 ± 0.068	0.875 ± 0.118	0.951 ± 0.072	1.000 ± 0.000	0.978 ± 0.039
